# Continuous Sustainable Production of Biobased Multicomponent Enhanced Resin for SLA 3D Printing

**DOI:** 10.1021/acsmaterialsau.5c00014

**Published:** 2025-03-20

**Authors:** Vojtěch Jašek, Otakar Bartoš, Veronika Lavrinčíková, Jan Fučík, Silvestr Figalla, Eliška Kameníková, Radek Přikryl

**Affiliations:** † Institute of Materials Chemistry, Faculty of Chemistry, Brno University of Technology, Brno 61200, Czech Republic; ‡ Institute of Environmental Chemistry, Faculty of Chemistry, Brno University of Technology, Brno 612 00, Czech Republic

**Keywords:** biobased, reactive diluent, curable resin, stereolithography, 3D printing

## Abstract

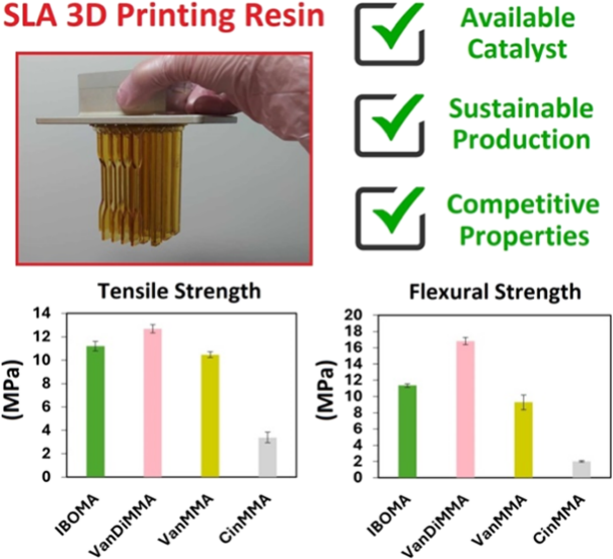

This work focuses on biobased reactive diluents’ synthesis, continuing with optimized oil-based resin precursor production. Our approach introduces vanillin methacrylate (VanMMA), cinnamyl methacrylate (CinMMA), and vanillyl dimethacrylate (VanDiMMA) synthesis using methacrylic anhydride. The introduced approach involves an innovative and available catalyst, potassium acetate, which possesses much suitable potential compared with the usually used 4-dimethylaminopyridine (DMAP). Moreover, we separated the formed secondary product, methacrylic acid (MA), and used it to modify rapeseed oil to prepare a curable thermoset. All synthesized products were structurally verified via complex cross-analysis (NMR, ESI-MS, and FTIR). The reactive systems were mixed to form a multicomponent mixture appropriate for stereolithography (SLA) and 3D printing. It was found that VanDiMMA exhibited comparable diluting properties to the commercially available and used compound, isobornyl methacrylate (IBOMA), while achieving better mechanical, thermo-mechanical, and thermal properties than IBOMA. VanDiMMA-containing SLA resin reached a tensile strength of 12.7 ± 0.3 MPa, a flexural strength of 16.8 ± 0.4 MPa, a storage modulus of 570 MPa at 30 °C, a glass-transition temperature of 83.7 °C, and the heat-resistant index of 169.5 °C.

## 1 Introduction

Stereolithography (SLA) is a practical, attractive, and popular technique for fabricating unique objects and prototypes for numerous purposes.^1−4^ Specific tools and parts,^2^ defined porous materials applied for heterogeneous catalysis,^3^ or various medicinal or conducting hydrogels^4,6^ are increasingly produced by SLA. This method involves many essential advantages. SLA provides exceptionally detailed printing, a key factor in the prototype’s fabrication.^5^ The precision level of the produced objects’ quality is incomparable with other 3D printing approaches, such as fused deposition modeling (FDM).^7,8^ Due to its exceptional versatility, many specific catalyst–supporting matrices are widely fabricated using SLA. The heterogeneous catalytic systems involve SLA-printed porous layers with defined pore shapes and sizes.^9,10^ This printed object is usually produced by SLA and then carbonized at increased temperatures (up to 1000 °C).^11^ Then, the carbonized chemically inert carrier can be coated with specific materials or systems exhibiting particular catalytic activity, such as metals,^12^ inorganic compounds,^13^ or specific multicomponent functional systems.^14^ The SLA-based porous heterogeneous catalysts commonly serve continuous-flow applications and chemical processes.^15^ The dental composite fabrication segment relies on stereolithography since the precise execution of modeled dental implants requires high manufacturing standards.^16,61^ Regarding dental applications, SLA exhibits many advantages in this field. Next to the detailed object fabrication, several suspensions and various heterogeneous dispersions can be manufactured by SLA. Since the dental industry incorporates particular inorganic systems such as silicocarnotite or ZrO_2_, additive manufacturing represents the optimal approach for this utility.^57,58,62^ Additive manufacturing possesses several benefits for aerospace applications. This commercial segment requires products with specific properties such as lightweight structure, efficient customization, shape recovery, or precise working micromechanism. Also, SLA 3D printing hardware does not require excessive working space; therefore, this approach can benefit particular space-limited aerospace setups.^59,60^

Several materials are used as reactive precursors for SLA-formed resins. Radically initiated polymerization is stereolithography’s most common chemical approach.^17^ The acrylates and methacrylates are popular and frequently used in this field.^18,19^ Usually, the systems that can form either brittle and rigid materials or elastic and flexible resins, depending on the chosen chemical modification, are selected for the precursors’ preparation.^20^ Several ethers,^21^ esters,^22^ amines,^23^ or thiols^24^ are functionalized to be photoinitially cured and form printed products. The common ether-based resin precursors from fossil sources are (poly)ethylene glycol acrylates/methacrylates such as PEGDA (poly(ethylene glycol) diacrylate).^25,26^ Nowadays, countless alternatives from renewable or recycled sources are introduced, investigated, and compared with high-performing fossil-based precursors.^27^ From the sustainability and scalability viewpoint, materials based on available resources (such as triacylglycerides, lignin derivatives, and natural molecules) are preferred and considered.^27^ Several modified triacylglycerides (TAGs) have been observed in the literature, such as acrylated/methacrylated oils,^28,29^ cross-linked epoxidized oils,^30^ and TAGs combined with diacids possessing various lengths from C6 to C32.^31^ The curable vegetable oils often possess high viscosity levels (1000–5000 mPa·s)^32^ and poor mechanical and thermo-mechanical properties (1–5 MPa tensile strength, 100–1000 MPa storage modulus, or 25–60 °C glass-transition temperature).^31,32^ Therefore, many reactive diluents enhance the overall properties of oil-based resins and are a part of additive manufacturing.^36^ Different structural reactive compounds modifying rheology,^33^ mechanical properties,^34^ or thermal stability^35^ are mixed with the oil-based matrixes. Namely, 4-acryloylmorpholine (ACMO),^37^ isosorbide dimethacrylate (IM, ISDMMA),^38,39^ isobornyl acrylate of methacrylate (IBOA, IBOMA),^40,41^ and ethylene glycol diacrylate (EGDA)^42^ are commonly used reactive diluents for commercial applications. The monofunctional compounds (ACMO, IBOA, IBOMA) are usually low-viscous, and their primary purpose is to modify the rheological profile.^37,40,41^ The multifunctional compounds (IM/ISDMMA or EGDA) increase the eventual products’ hardness, brittleness, and mechanical robustness.^38,39,42^

The reported production approaches leading to reactive oil-based precursors and reactive diluents from renewable sources do not deal with the nonsustainability of the chemical process. Acyl halides,^43^ carboxylic acids’ anhydrides,^44^ or specific reactive species such as glycidyles^45^ often produce undesired secondary products depending on the used nucleophile. Hydrochloride acid is a secondary product for modifications using acyl chlorides,^43^ and particular carboxylic acids (acrylic/methacrylic acid) are formed in case of methacrylate application.^44^ Also, toxic or expensive catalysts are often used to produce biobased systems for material applications such as 4-dimethylaminopyridine (DMAP)^46^ or Lipozyme RM IM (*Rhizomucor miehei* lipase).^47^ The presented approaches often involve unscalable steps and do not deal with excessive waste disposal. In the case of acyl chloride usage, the formed hydrochloric acid prevents the production increase.^43^ Methacrylic acid, formed during the reaction involving methacrylic anhydride, is usually washed and neutralized in published papers and articles.^38,46,47^ Although several published investigations present intriguing strategies for producing biobased materials appropriate for SLA, the described processes lack engineering aspects.

This article presents the synthesis of reactive diluents from renewable sources: cinnamyl alcohol (produced from storax),^48^ vanillin, and vanillyl alcohol (produced from lignin waste).^49^ Methacrylic anhydride is used as a nucleophile donor for the chemical modification of the mentioned alcohols. We used an alternative catalyst to DMAP, which is commonly mentioned in the literature.^38,46^ Potassium acetate (KAc) catalyzed the reaction. This compound can be manufactured from biobased acetic acid and is significantly cheaper than DMAP. The formed secondary product, methacrylic acid (MA), was isolated from the reaction mixture. Therefore, potential waste disposal is minimized. Continually, the obtained MA was used to modify the rapeseed oil. This substrate was epoxidized in the first step. Then, the oxirane rings within the epoxidized oil structure were exposed to the separated MA-producing methacrylated rapeseed oil (MRO). MRO was eventually mixed with synthesized reactive diluents: cinnamyl alcohol methacrylate (CinMMA), vanillin methacrylate (VanMMA), and vanillyl dimethacrylate (VanDiMMA). The rheological profile changes, complex mechanical and thermo-mechanical investigation, and thermal stability of formed curable systems were explored. SLA 3D printing was used to test the specimens’ production, proving the synthesized systems’ suitability for stereolithography.

## 2 Experimental Section

### 2.1 Materials

The biobased entering reactants for reactive diluents’ production, vanillin, vanillyl alcohol, and cinnamyl alcohol, were obtained from Sigma-Aldrich. The rapeseed oil used for the modified oil preparation was purchased from Fichema Ltd. Methacrylic anhydride MAAH (94%) was obtained from Visiomer©. Other chemicals for the reactive diluent syntheses, sodium hydroxide (NaOH) for isolation, potassium acetate (KAc) for catalysis, and sodium sulfate (Na_2_SO_4_) for drying were obtained from PENTA Chemicals Ltd. (Czech Republic). The reactants for modified oil synthesis, hydrogen peroxide (H_2_O_2_, 30%), formic acid (HCOOH, 98%), potassium iodide (KI, 99%), sodium thiosulfate (Na_2_S_2_O_3_ 99%, anhydrous), triethylamine (TEA, 99%), and ethyl acetate (EtAc, 99%) were obtained from Sigma-Aldrich. The polymerization inhibitor, Genorad 26, was purchased in UL Solutions. The additional substances required for the analyses and experiments, BAPO (photoinitiator, phenylbis(2,4,6-trimethylbenzoyl)phosphine oxide), and d-chloroform (solvent for NMR analyses, CDCl_3_) were all purchased also from Sigma-Aldrich.

### 2.2 Structural Verification Methods

Nuclear magnetic resonance (NMR) served to confirm the products’ structures. The instrumentation was a Bruker Avance III 500 MHz (Bruker, Billerica, MA). The measuring frequency was 500 MHz for ^1^H NMR and 126 MHz for ^13^C NMR. The measuring temperature was 30 °C, and d-chloroform (CDCl_3_) served as a solvent. Tetramethylsilane (TMS) served as an internal standard. The chemical shifts (δ) are expressed in parts per million (ppm) units, referenced by a solvent. Coupling constant (*J*) is expressed with frequency unit (Hz) with coupling expressed as *s*-singlet, *d*-doublet, *t*-triplet, *q*-quartet, *p*-quintet, and *m*-multiplet.

Electrospray mass spectrometry (ESI-MS) was used for the structural cross-analysis. The applied instrumentation was Bruker EVOQ LC-TQ. Product scan spectra were obtained by fragmentation of the following [M + H]^+^ precursor ions detailed in the synthesis section for every product. Collision energy spread (5–20 eV) improved the collected MS/MS data quality. Furthermore, the obtained mass spectra agree with their in silico prediction by CFM-ID 4.0,^50^ which also proposed the product ion structure for the most intensive masses.

Fourier-transform infrared spectroscopy (FTIR) participated in the cross-analysis of products’ structure verification. Analyses were performed using a Bruker Tensor 27 (Billerica, MA) and the attenuated total reflectance (ATR) method. Diamond served as the dispersion component. A diode laser was the irradiation source in this spectroscope. The Michelson interferometer was used to quantify the signal. Spectra comprised 32 total scans with a measurement resolution of 2 cm^–1^.

### 2.3 Oil Characterization Methods

Acid value (A.V.) quantifies acidic functional groups. The applied norm for A.V. was SN EN ISO 660.

Oxirane oxygen content (OOC) quantifies the percentage amount of cyclic bonded oxygen as oxidation products. The method’s principle is the nucleophilic substitution reaction of hydrobromic acid (HBr) and epoxy functional groups. The proton opens the epoxy group’s cyclic structure, and the nucleophile (Br^–^) attacks the formed carbocation. The sample (0.1–0.3 g) is added to the titration flask. Next, 10 mL of 99% w/w acetic acid is added. This solution is enriched with 5 drops of crystal violet (indicator) and titrated by 0.1 M HBr in acetic acid. The calculation of oxirane oxygen content is written in eq 1:
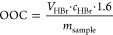
1where OOC represents oxirane oxygen content (%), *c*_HBr_ refers to the molar concentration of the titration solution (mol/dm^3^), *V*_HBr_ signs the volume of titration of the solution for sample (cm^3^), and *m*_sample_ is the weight of the measured sample (g).

### 2.4 Synthesis of Biobased Reactive Diluents

The particular biobased alcohol was transferred into a 1000 mL round-bottom flask (1 mol of each entering reactant prior to the reaction). The methacrylation agent, MAAH, was introduced into the alcohol in equimolar amounts to the free hydroxyl groups: vanillin and cinnamyl alcohol methacrylation reaction (1 mol of MAAH) and vanillyl alcohol methacrylation reaction (2 mol of MAAH). The reaction solution was heated to 80 °C. This reaction temperature was determined as optimal for such reactions based on our previously published works.^39,51^ The catalyst, KAc, was added to each mixture (0.01 mol %). The reaction lasted 8 h at 80 °C. After the reaction time, the secondary product, methacrylic acid (MA), was distilled. The polymerization inhibitor Genorad 26 was added to the mixture (5 drops), and the solution was distilled at 100 °C and 1 kPa. The remaining acidity of the product solution was neutralized by NaOH in water solution, and the nonacidic solution was dried over Na_2_SO_4_. The distilled MA and the synthesized products were structurally analyzed via NMR, ESI-MS, and FTIR.

#### 2.4.1 Vanillin Methacrylate (VanMMA)

^1^H NMR (500 MHz, CDCl_3_) δ 9.94 (s, 1H), 9.92 (s, 0H), 7.51–7.43 (m, 2H), 7.28–7.20 (m, 1H), 6.37 (p, *J* = 1.0 Hz, 1H), 5.78 (p, *J* = 1.5 Hz, 1H), 3.88 (s, 3H), 2.06 (dd, *J* = 1.6, 1.0 Hz, 3H).

^13^C NMR (126 MHz, CDCl_3_) δ 191.18, 164.93, 152.30, 145.42, 135.37, 135.32, 128.02, 124.86, 123.62, 111.04, 56.27, 56.21, 18.49.

ESI-MS fragmentation spectrum (C_12_H_12_O_4_) spectrum calc. [M + H]^+^ 220.22 *m*/*z*, found 220.0 *m*/*z*.

FTIR spectrum absorption wavenumber intervals: C–H stretch, 3000–2840 cm^–1^; C=O (ester) stretch, 1750–1735 cm^–1^; C=C stretch, 1662–1626 cm^–1^; C–O (ester) stretch, 1210–1163 cm^–1^; C=C bend, 840–790 cm^–1^.

#### 2.4.2 Cinnamyl Methacrylate (CinMMA)

^1^H NMR (500 MHz, CDCl_3_) δ 7.43–7.36 (m, 2H), 7.36–7.29 (m, 2H), 7.33–7.23 (m, 1H), 6.68 (dt, *J* = 15.8, 1.5 Hz, 1H), 6.33 (dt, *J* = 15.9, 6.4 Hz, 1H), 6.16 (dq, *J* = 2.0, 1.0 Hz, 1H), 5.59 (p, *J* = 1.6 Hz, 1H), 4.82 (dd, *J* = 6.3, 1.4 Hz, 2H), 1.98 (dd, *J* = 1.6, 1.0 Hz, 3H), 1.61–1.53 (m, 1H).

^13^C NMR (126 MHz, CDCl_3_) δ 167.22, 136.36, 136.30, 134.06, 128.62, 128.06, 126.64, 125.66, 123.37, 65.27, 18.35.

ESI-MS fragmentation spectrum (C_13_H_14_O_2_) spectrum calc. [M + H]^+^ 202.25 *m*/*z*; found 203.0 *m*/*z*.

FTIR spectrum absorption wavenumber intervals: C–H stretch, 3000–2840 cm^–1^; C=O (ester) stretch, 1750–1735 cm^–1^; C=C stretch, 1662–1626 cm^–1^; C–O (ester) stretch, 1210–1163 cm^–1^; C=C bend, 840–790 cm^–1^.

#### 2.4.3 Vanillyl Dimethacrylate (VanDiMMA)

^1^H NMR (500 MHz, CDCl_3_) δ 7.05 (d, *J* = 7.9 Hz, 1H), 7.00–6.96 (m, 2H), 6.35 (q, *J* = 1.1 Hz, 1H), 6.16 (dq, *J* = 2.0, 1.0 Hz, 1H), 5.75 (p, *J* = 1.6 Hz, 1H), 5.60 (p, *J* = 1.6 Hz, 1H), 5.17 (s, 2H), 3.83 (s, 3H), 2.07 (dd, *J* = 1.5, 1.0 Hz, 3H), 1.98 (dd, *J* = 1.6, 1.0 Hz, 3H).

^13^C NMR (126 MHz, CDCl_3_) δ 167.31, 165.53, 151.42, 139.99, 136.34, 135.73, 135.03, 127.38, 126.03, 123.02, 120.66, 112.58, 86.24, 66.23, 56.12, 18.56, 18.47.

ESI-MS fragmentation spectrum (C_16_H_20_O_2_) spectrum calc. [M + H]^+^ 292.33 *m*/*z*, found 291.0 *m*/*z*.

**Figure 1:**
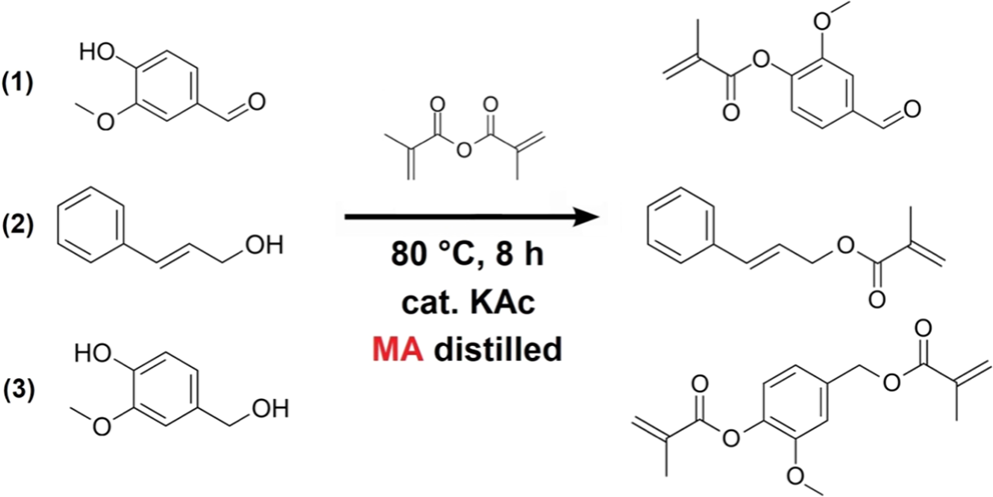
Reaction scheme of reactive diluents’ synthesis. (1) VanMMA synthesis, (2) CinMMA synthesis, and (3) VanDiMMA synthesis.

FTIR spectrum absorption wavenumber intervals: C–H stretch, 3000–2840 cm^–1^; C=O (ester) stretch, 1750–1735 cm^–1^; C=C stretch, 1662–1626 cm^–1^; C–O (ester) stretch, 1210–1163 cm^–1^; C=C bend, 840–790 cm^–1^ (Figure 1).

#### 2.4.4 Methacrylic Acid (MA)

^1^H NMR (500 MHz, CDCl_3_) δ 11.68 (s, 1H), 6.26 (dd, *J* = 1.5, 1.0 Hz, 1H), 5.68 (p, *J* = 1.6 Hz, 1H), 1.96 (dd, *J* = 1.6, 1.0 Hz, 3H).

^13^C NMR (126 MHz, CDCl_3_) δ 174.28, 138.26, 126.09, 17.93.

ESI-MS fragmentation spectrum (C_4_H_6_O_2_) spectrum calc. [M–H]^−^ 85.06 *m*/*z*, found 85.1 *m*/*z*.

FTIR spectrum absorption wavenumber intervals: O–H stretch, 3550–3200 cm^–1^; C–H stretch, 3000–2840 cm^–1^; C=C stretch, 1662–1626 cm^–1^; C–O (acid) stretch, 1210–1163 cm^–1^; C=C bend, 840–790 cm^–1^.

### 2.5 Epoxidation of Rapeseed Oil

**Figure 2:**
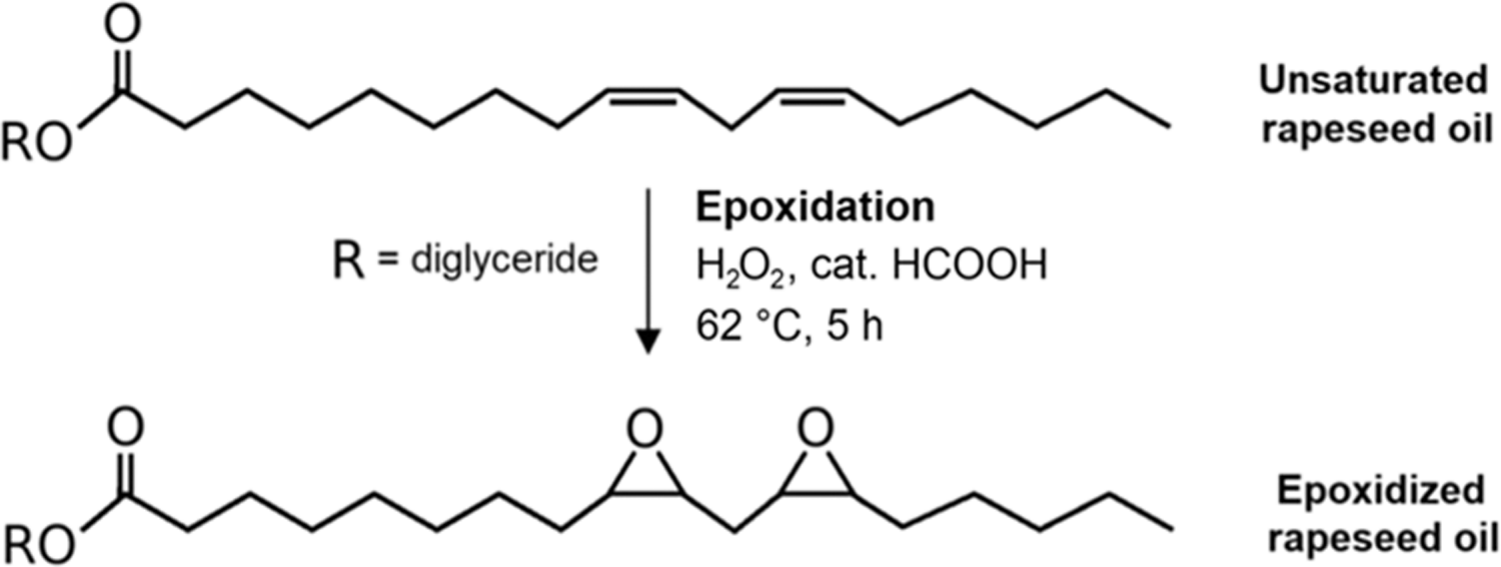
Reaction scheme of epoxidized rapeseed oil synthesis (ERO).

Rapeseed oil (912 g), 30% hydrogen peroxide (1.4 mol), and formic acid as a catalyst (0.25 mol) were added to the reactor (2000 mL) equipped with a shaft stirrer and a heating jacket. The reaction scheme is illustrated in Figure 2. The oil was initially heated to 43 °C due to the expected exothermic reaction. Then, the mixture containing H_2_O_2_ and HCOOH was poured into the reactor and homogenized. Immediately, the reaction was set to 62 °C. The reaction was performed for 5 h. The samples were obtained regularly during the reaction, and the volumetric analyses (I.V. and OOC) were performed (the samples were O/W emulsions containing partially epoxidized oil and H_2_O_2_ + HCOOH water solution excess, and the separation of phases using a centrifuge was performed). Epoxidized oil was separated from the water phase. Next, the reduction of peroxides was performed using potassium iodide as a reduction agent. The eventual epoxidized oil purification involved the separation of the formed iodine (as a product of the reduction) by the sodium thiosulfate water solution. Once the equivalent amount of Na_2_S_2_O_3_ was added to reduce I_2_, the emulsion turned white, indicating the disappearance of iodine (I_2_ containing oil was dark brown). Finally, a centrifuge separated the purified oil from the water–salt solution. The epoxidized oil was eventually mixed with Na_2_SO_4_ to remove residual water. The epoxidized rapeseed oil (ERO) was structurally verified.

#### Epoxidized Rapeseed Oil (ERO)

^1^H NMR (500 MHz, CDCl_3_) δ 4.33–4.12 (m, 4H), 3.19–2.85 (m, 3H), 2.31 (tt, *J* = 7.4, 2.6 Hz, 5H), 1.66–1.17 (m, 60H), 0.89 (dt, *J* = 11.4, 7.1 Hz, 7H).

FTIR spectrum absorption wavenumber intervals: C–H stretch, 3000–2840 cm^–1^; C=O (ester) stretch, 1750–1735 cm^–1^; C–O (ester) stretch, 1210–1163 cm^–1^; C–O–C stretch, 840–790 cm^–1^.

### 2.6 Epoxidized Oil Methacrylation Using Distilled Methacrylic Acid

**Figure 3:**
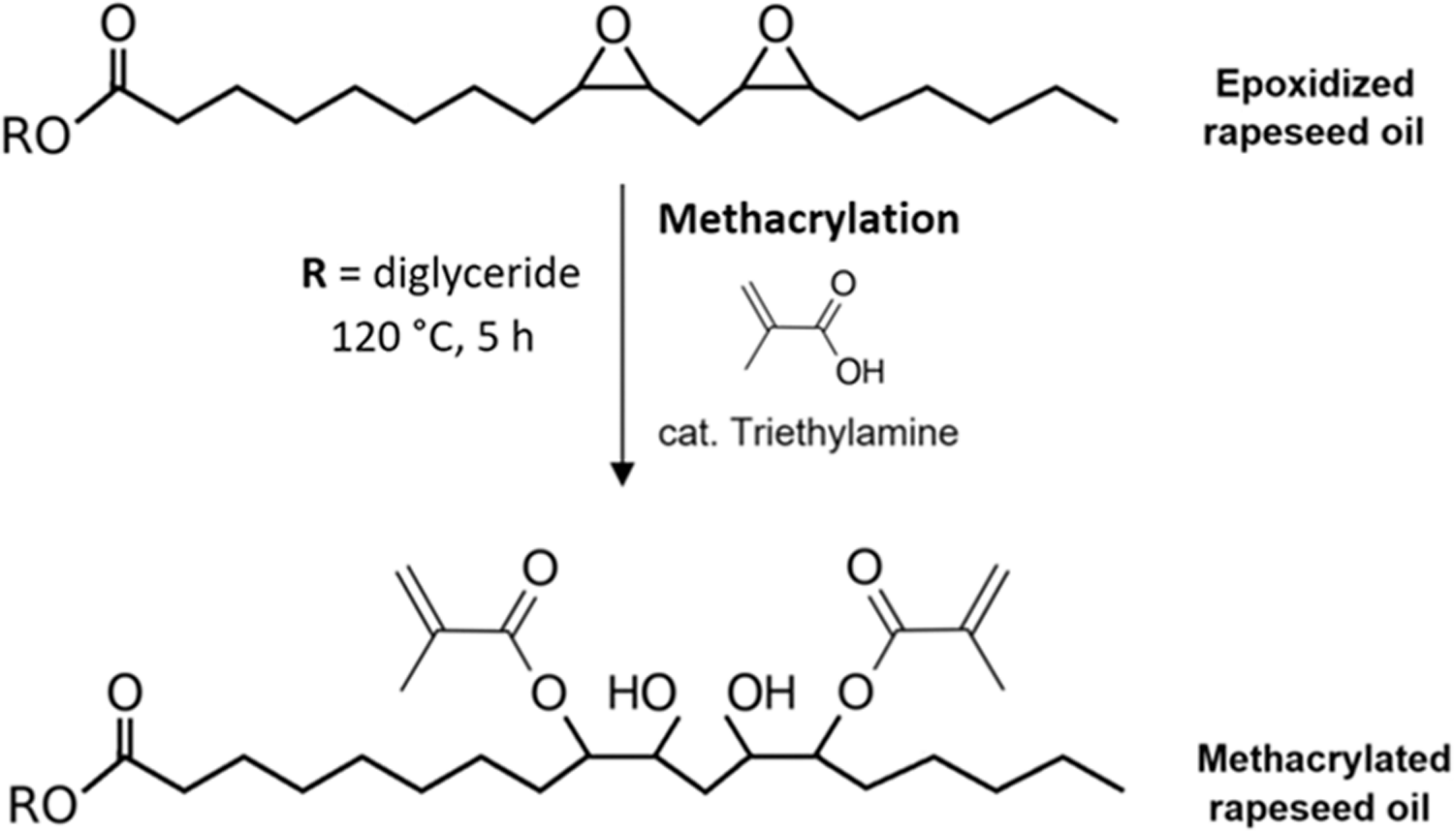
Reaction scheme of methacrylated rapeseed oil synthesis (MRO).

The reaction molar ratio was equimolar (OOC molar mass to methacrylic acid molar mass). The 1 mol equivalent of oxirane oxygen content of the OOC (253.0 g of epoxidized rapeseed oil) was mixed with methacrylic acid (1 mol). The reaction scheme is illustrated in Figure 3. The reaction solution with all components was heated to 120 °C. The catalyst (TEA, 0.05 mol) was poured into the mixture. The nucleophilic substitution (methacrylation) was performed for 5 h. The reaction batch purification involved adding ethyl acetate (to form 50 wt % solution with product mixture) and residual methacrylic acid neutralization. NaOH, equivalent to methacrylic acid, was dissolved in distilled water and poured into the product mixture. The formed emulsion was centrifuged, and the water phase containing the formed methacrylic salts was separated. Eventually, the nonacidic product was twice extracted with pure distilled water to remove residual salts and catalysts. Lastly, the solvent (EtAc) was distilled. The formed methacrylate rapeseed oil was characterized (MRO).

#### Methacrylated Rapeseed Oil (MRO)

^1^H NMR of methacrylated rapeseed oil (500 MHz, CDCl_3_) δ 6.16–6.09 (m, 2H), 5.58 (q, *J* = 2.1 Hz, 2H), 4.29 (dd, *J* = 11.9, 4.3 Hz, 2H), 4.18–4.08 (m, 3H), 2.30 (tt, *J* = 7.5, 2.3 Hz, 6H), 2.08–1.88 (m, 9H), 1.80–1.11 (m, 60H), 0.88 (tt, *J* = 7.1, 1.6 Hz, 7H).

FTIR spectrum absorption wavenumber intervals: O–H stretch, 3550–3200 cm^–1^; C–H stretch (alkene), 3100–3000 cm^–1^; C–H stretch (alkene), 3000–2840 cm^–1^; C=O (ester) stretch, 1750–1735 cm^–1^; C=C stretch, 1662–1626 cm^–1^; C–O (ester) stretch, 1210–1163 cm^–1^; C=C bend, 840–790 cm^–1^.

### 2.7 Rheological Modification Investigation

The rheological behavior was investigated (TA Instruments rheometer AR-G2) to investigate the rheological profile of prepared reactive diluent mixtures with methacrylated rapeseed oil. The primary purpose of such compounds is to decrease the target system’s apparent viscosity. We prepared the mixtures of MRO with each reactive diluent in different weight mass compositions (0, 5, 10, 15, 20, 25, 30 wt % of reactive diluent in MRO). These systems were measured at the following conditions: 500 μL quantity of sample, the Peltier platform and cone–plate geometry (40 mm, 2° angle), the shear rate of 10 s^–1^, and the temperature set to 25 °C.

### 2.8 SLA 3D Printing of the Prepared Resins

The printability of the mixtures was determined using a PRUSA SL1 (Prusa Research s ro., Praha, Czech Republic) 3D printer by printing test specimens for mechanical testing. All printed systems contained 25 wt % of each reactive diluent and 1 wt % of photoinitiator, BAPO. The print settings were as follows: the first 10 layers’ exposure time was 35 s, while the exposure time for all subsequent layers was 25 s. The thickness of the cured layer was defined as 50 μm. The test specimens were printed with automatically generated supports along with a raft to ensure good adhesion of the samples to the build platform. After printing, the samples were cleaned with isopropanol and postcured under a 405 nm LED light for 30 min.

### 2.9 Mechanical Properties of 3D-Printed Resins

The mechanical properties of prepared mixtures were determined using tensile and flexural testing. Both tests were performed on a Zwick Z 010 testing machine (ZwickRoell GmbH & Co., Ulm, Germany) equipped with a 500 N load cell. The tensile test was performed according to the CSN EN ISO 527 standard using standardized double-paddle specimens (dogbones 5A) with a 4 × 2 mm cross-sectional area. The test speed was set to 5 mm·min^–1^.

For the three-point flexural test, rectangular specimens with dimensions of 80 × 10 × 4 mm were printed according to the CSN EN ISO 178 standard (by which the test was conducted). The loading nose and support radius were 5 mm, with a support span of 64 mm. The test speed was set to 10 mm·min^–1^.

### 2.10 Thermo-Mechanical Properties of 3D-Printed Resins

The study of prepared materials’ viscoelastic properties and determination of glass-transition temperature (*T*_g_) were obtained by dynamic mechanical analysis (DMA). We used the analyzer DMA 2980 from TA Instruments (New Castle, DE). Testing specimens with typical dimensions of 60 × 10 × 2 mm were fabricated by an SLA printer. The curing process lasted 30 min. The specimens were mounted into dual cantilever geometry and subjected to a cyclic deformation with 10 μm amplitude and 1 Hz frequency. The samples were heated from the laboratory to 120 °C at a heating rate of 3 °C·min^–1^.

The heat resistance was studied by thermogravimetric analysis (TGA). TGA analysis was performed on a TGA Q500 from TA Instruments (New Castle, DE). The degradation process of a sample (10–15 mg) was monitored via the following heating conditions: equilibration at 40 °C; heating to 600 °C at a heating rate of 10 °C/min under N_2_; 10 min at 600 °C under air atmosphere. The heat-resistant index was obtained from the proposed eq 2:
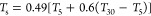
2where *T*_s_ is heat-resistant index (°C), *T*_5_ stands for temperature at 5% of mass loss (°C), and *T*_30_ represents temperature at 30% of mass loss (°C).

We also studied the volatility of the synthesized and referenced reactive diluents. The experiments were designed as isothermal TGA measurements, monitoring the mass decrease over the measurement time. The analyses were performed under the following conditions: equilibration at 50 °C under N_2_ and maintenance for 360 min.

## 3 Results and Discussion

### 3.1 Reactive Diluents’ Synthesis

The continual process resulting in multicomponent SLA 3D printing resins begins with the synthesis of reactive diluents. This process uses MAAH as a nucleophile donor to modify free hydroxyl groups (see Figure 1), producing reactive compounds with the secondary product, methacrylic acid. Potassium acetate served as a previously verified appropriate catalyst for this reaction. The GC-MS study performed in our previous investigation verified the quantitative conversions.^39^ The results of this synthesis at an equimolar reactant ratio are summarized in Table 1. The product yields range from 71 to 75%, which is sufficient. The available literature sources report a wide range of methacrylated products derived using methacrylic acid or anhydride (reported 54% yield,^63^ 17%,^64^ 56% yield,^65^ 66.5%^38^). Separating secondary products is crucial for sustainability and more efficient wastewater management. Since most disposable MA is separated, the total waste produced during the process is decreased significantly (the decrease is equal to the separated byproduct mass content). This phenomenon was observed in previously published works, and purification losses cause yield decrease.^38^ In the presented work, the experimental yield for the equimolar MAAH content reached 66% of the theoretical. Our yields are slightly higher, while the secondary product was also separated from the reaction mixture. The majority of distilled MA certainly helped with the yield increase; however, the production could be improved with the quantitative separation of formed MA. This step would prevent the washing/neutralization step from decreasing the yield due to process losses. Maintenance of the MA in the mixture can improve the reaction’s effectiveness. The extraction-involving process requires additional working steps involving supportive solvent removal or the application of partially acidic products in appropriate processes, such as pultrusion. The future ambitions should focus either on maximal MA separation or on maintaining its presence in the mixture.

**Table 1:** Results of Reactive Diluents’ Synthesis

**reactive diluents’ synthesis**					
**product**	entering reactant (mol)	MAAH (mol)	MA (distilled) (mol)	product yield (%)	distilled MA yield (%)
**VanMMA**	1.0	1.0	0.74	75.2	74.0
**CinMMA**	1.0	1.0	0.76	72.3	76.0
**VanDiMMA**	1.0	2.0	1.59	71.5	79.5

**Figure 4:**
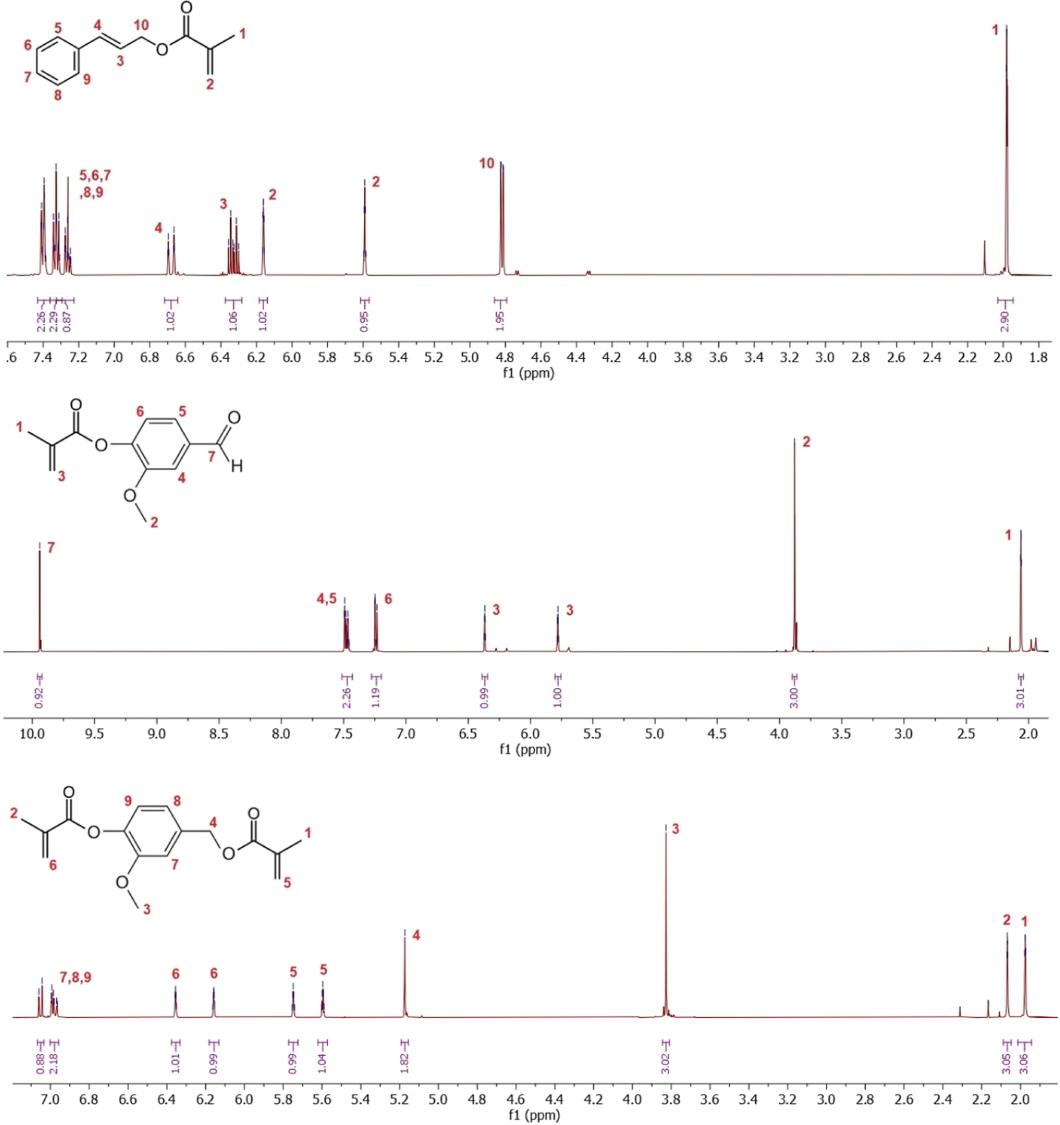
^1^H NMR spectra of the synthesized reactive diluents. Top, cinnamyl methacrylate (CinMMA); middle, vanillin methacrylate (VanMMA); bottom, vanillyl dimethacrylate (VanDiMMA).

We illustrate the ^1^H NMR spectra of the synthesized reactive diluents in Figure 4. The rest of the cross-structural analysis involving ^13^C NMR, ESI-MS, and FTIR is a part of Supplementary. The illustrated ^1^H NMR spectra contain all of the expected signals according to the prediction. The expected compounds’ structures were verified together with the other confirmation analyses. All illustrated spectra (Figure 4) contain minor impurities at lower chemical shift intervals (2.2–2.0 ppm). These impurities represent the methylene proton of residual methacrylic acid in the product. However, the measured acidity of all synthesized compounds was negligible (<1 mg KOH/g); therefore, the overall purity of synthesized reactive diluents was sufficient (>99% purity) based on the acidic value.

### 3.2 Continual Methacrylated Oil Synthesis

**Figure 5:**
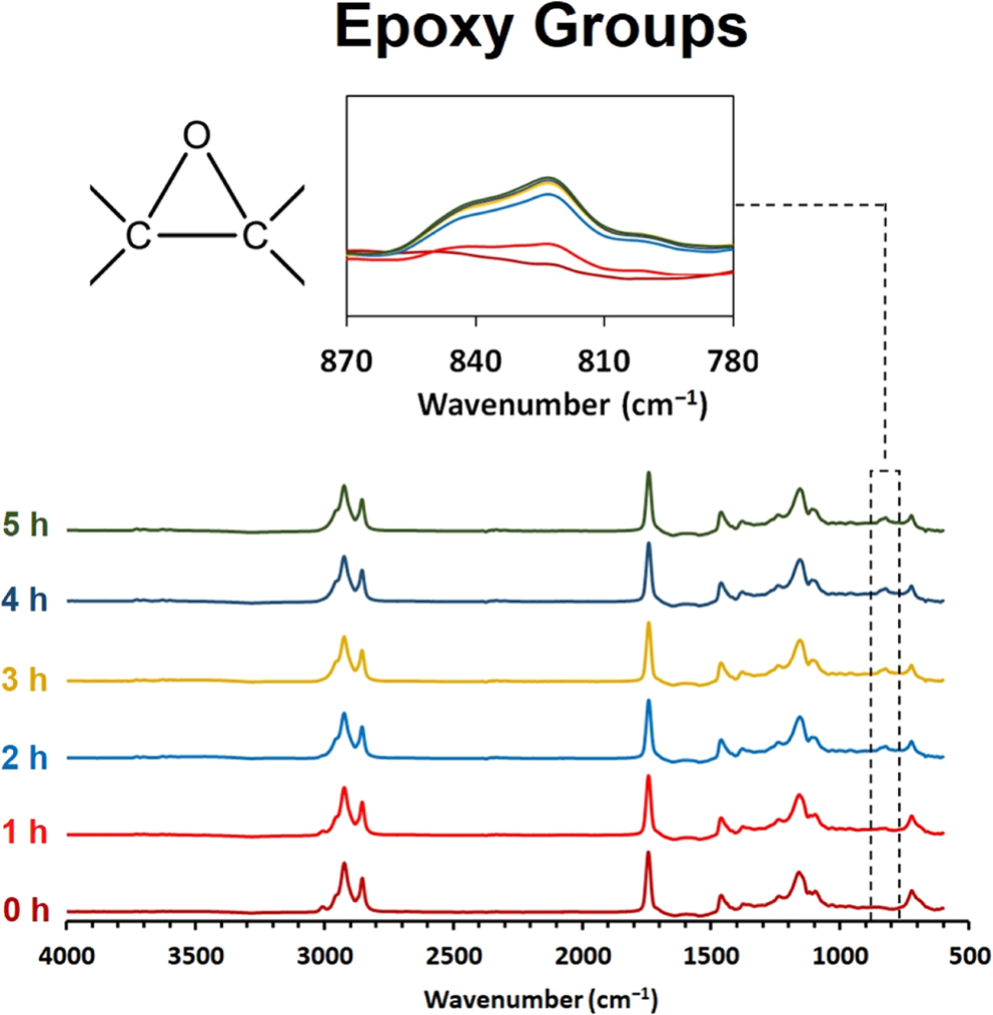
Rapeseed oil’s FTIR epoxidation kinetics study via FTIR during the reaction.

Epoxidized vegetable oils are widely used for highly biobased curable resins since these entering materials are available, easily produced, and obtainable across the world. Epoxy functional groups are enormously reactive and undergo several nucleophilic substitutions. We used methacrylic acid as a nucleophile, modifying the epoxidized oil’s structure. This approach ensures a curable precursor’s production containing serious content from renewable sources. The rapeseed oil epoxidation was monitored via volumetric analysis (OOC determination) and FTIR during the reaction. The determination of the OOC served as the basis for the online double bond conversion based on decreased alkenes in the oil structure. The results of volumetrically determined conversion are summarized in the combined Table 2. The analysis uncovered practically quantitative conversion of the present double bonds. We reached 97.5% conversion based on the OOC formation calculation. The calculation is explained in our previous work.^52^ The iodine value determined for the entering vegetable oil is calculated for the theoretical number of unsaturated bonds, and this value is recalculated for the oxirane weight content (represented in %). The theoretical OOC value is 7.11% for the I.V. of 125.5 g of I_2_/100 g (measured at the beginning of the experiment). The kinetics of the epoxidation are also illustrated in Figure 5 based on the FTIR investigation. We detail the characteristic C–O–C absorption segment at 870–780 cm^–1^. Based on the volumetrically determined results from Table 2 and the FTIR analyses from Figure 5, the reaction reached its equilibrium after 180 min of the reaction. Therefore, the set reaction time is unnecessary for the quantitative conversion of most present double bonds in the rapeseed oil’s structure. The double bond disappearance is also evident from the FTIR spectra at a wavenumber value of 3000 cm^–1^.

**Table 2:** Summarized Results of Rapeseed Oil’s Modifications

**modified rapeseed oil synthesis**					
**epoxidation**			**methacrylation**		
**reaction time (min)**	**OOC (%)**	**conversion fromOOC (%)**	**reaction time(min)**	**A.V.(mg KOH/g)**	**conversion fromA.V. (%)**
0	0	0.0	0	112.7	0.0
30	2.25	31.6	20	75.2	33.3
60	2.48	34.9	40	46.3	58.9
90	3.98	56.0	60	37.9	66.4
120	5.40	75.9	120	21.0	81.4
180	6.89	96.9	180	15.8	86.0
240	6.95	97.7	240	12.5	88.9
300	6.93	97.5	300	10.5	90.7

**Figure 6:**
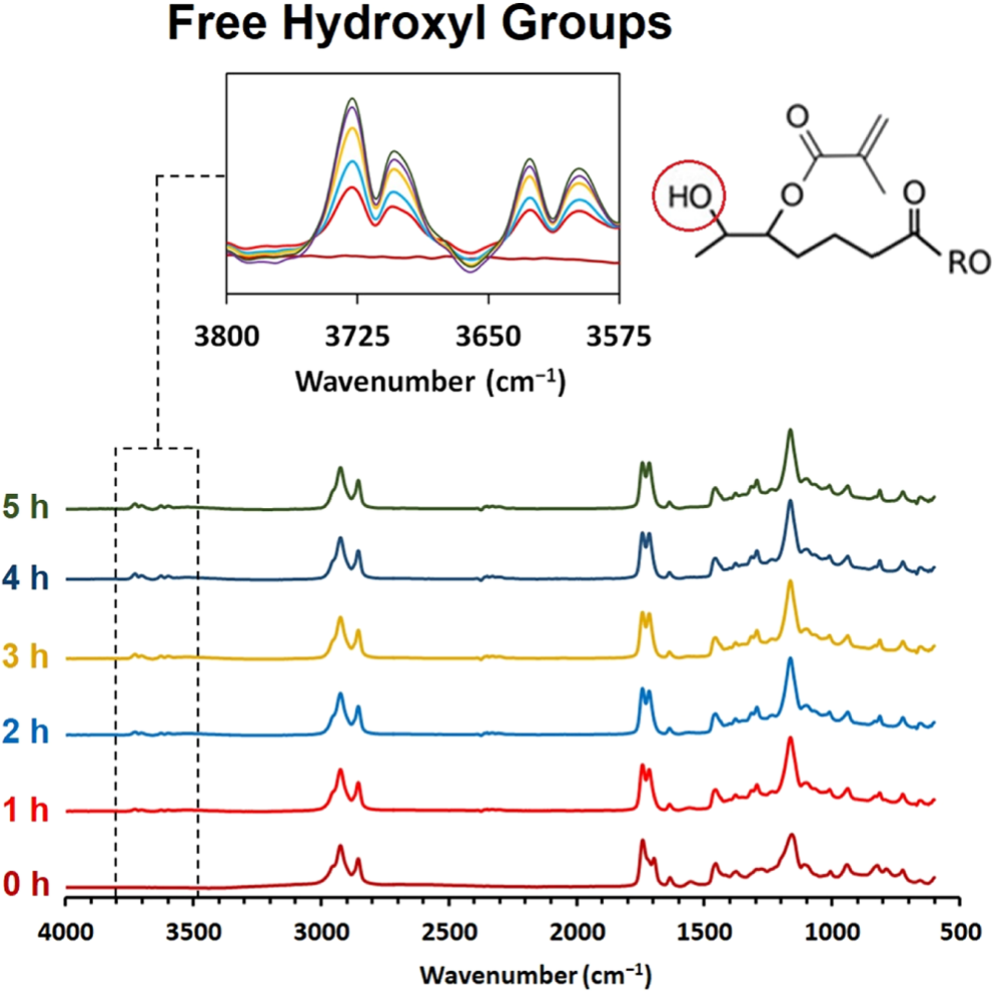
Rapeseed oil’s FTIR methacrylation kinetics study via FTIR during the reaction.

The following methacrylation of synthesized epoxidized oil is numerically summarized in Table 2, and the reaction study provided FTIR analysis during the nucleophilic substitution, as illustrated in Figure 3. Also, the structure verification of the separated MA from the previous reactive diluents’ synthesis is added to the modified rapeseed oil’s NMR spectra in Figure 6. The nucleophile substitution (methacrylation) was monitored by the acidity decrease during the reaction since the methacrylic acid possessed specific and measurable A.V. that was reacting during the modification. Therefore, the acidity of the reaction mixture should decrease. The eventual conversion based on the decreased acidic value reached 90.7%, which is a sufficient result. The published modified oil results reached values around 85%^66^ and 69–85%.^67^ Online FTIR also confirmed the nucleophile substitution since the unbonded hydroxyl groups occurred in the oil’s structure. This formation is a secondary effect of the methacrylation and the chemical oxirane ring’s opening. The conversion values reached have improved compared to our previous publication.^52^ The reaction temperature has the main effect on the improved conversion results. In our previous work, the methacrylation was performed at 95 °C and reached 23.08% conversion after 12 h. Our presented experiment achieved much better results at 120 °C in a shorter time. The reaction kinetics was also verified via FTIR. The region of free hydroxyl groups at 3800–3575 cm^–1^ exhibited the absorption signal occurrence and increase. This is a direct consequence of nucleophile substitution forming the hydroxyl group (Figure 3). The other confirmation of undergoing the reaction lies at 1700 cm^–1^, where the absorption peak rises as the reaction progresses. This signal refers to C=O (stretching) of forming methacrylate ester bond.

**Figure 7:**
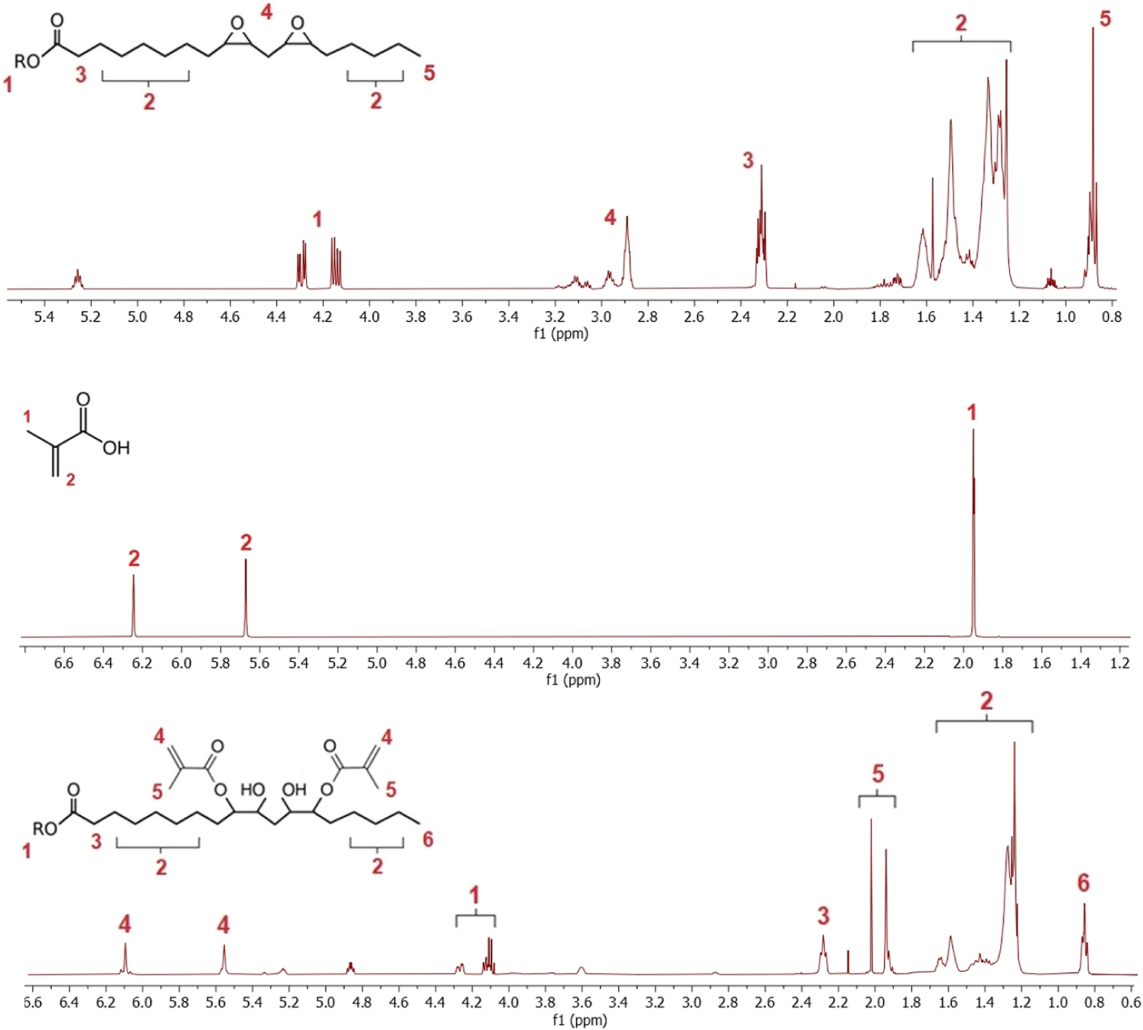
^1^H NMR spectra of the synthesized epoxidized oil, methacrylated oil, and distilled methacrylic acid. Top, epoxidized rapeseed oil; middle, distilled methacrylic acid (MA); bottom, methacrylated rapeseed oil.

The structural verifications of synthesized epoxidized oil, distilled methacrylic acid, and methacrylated oil are displayed in Figure 7. All measured signals were described according to the prediction, and the integrated values are a part of the Supplementary. The ^1^H NMR results are comparable to those interpreted in published articles in experimental spectra.^53^ The epoxidized oil’s NMR spectrum primarily uncovers the epoxy functional group occurrence in the carbon backbone (3.2–2.9 ppm). The methacrylic acid NMR spectrum did not reveal any possible impurities in the secondary product. Methacrylated oil’s NMR spectrum does not contain epoxy functional groups. At the same time, signals at 6.1 and 5.55 ppm confirm the presence of unsaturated curable functional groups.

### 3.3 Rheological Profile of Synthesized Systems

**Figure 8:**
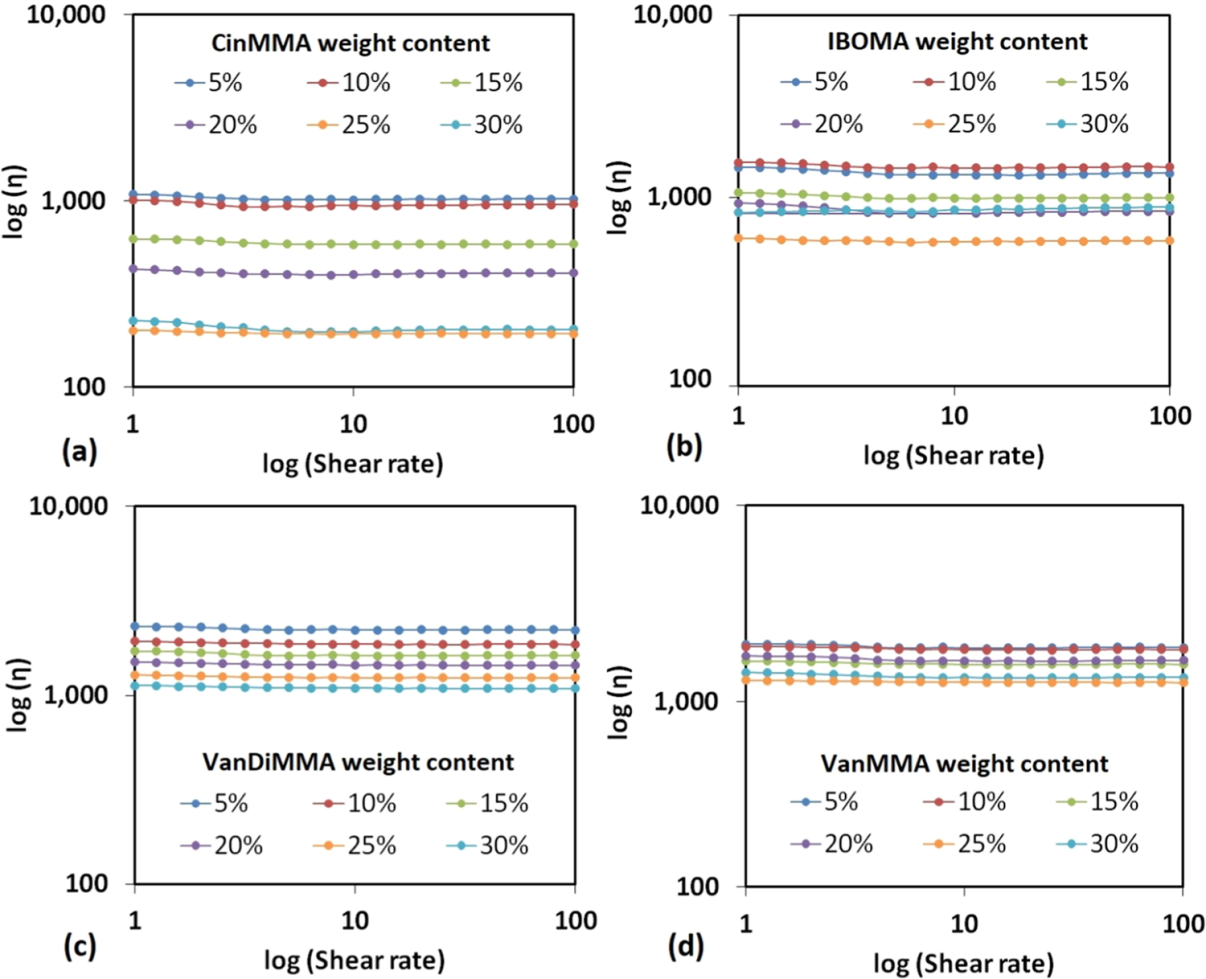
Rheological modification of the synthesized methacrylated oil by the produced reactive diluents. (a) Cinnamyl methacrylate (CinMMA), (b) isobornyl methacrylate (IBOMA), (c) vanillyl dimethacrylate (VanDiMMA), and (d) vanillin methacrylate (VanMMA).

The synthesized reactive diluents primarily modify the viscosity levels of targeted highly viscous systems to improve their processability and increase their field of usage. The regulation of the rheology profile is essential for several applications, including SLA 3D printing, since this utility’s maximally used curable systems cannot pass 5000 mPa·s based on the reported and published experience.^54^ We performed the viscosity measurement for our synthesized MRO and then the increasing weight content of the synthesized reactive diluents to investigate their rheological modifying properties. The graphical results illustrating the constant viscosity values with varying shear rates (confirming the Newtonian flow behavior) are summarized in Figure 8.

Based on the obtained results measured at 25 °C temperature, CinMMA succeeded in the highest viscosity decrease to 190 mPa·s at 30 wt % content in MRO. VanMMA and VanDiMMA resulted in practically the same (VanDiMMA ensured slightly lower viscosities); both reached around 1000–1200 mPa·s at around 30 wt % content in MRO. The diluting was successful in all studied cases since the 100% MRO exhibited a viscosity value of around 2700 mPa·s, signalizing a significant decrease in all studied cases. We also investigated the diluting properties of the commercially available compound, isobornyl methacrylate (IBOMA), to emphasize the comparison among the reactive diluents. IBOMA exhibited the viscosity of 580 mPa·s at 30 wt % content in MRO, which uncovers a better diluting character than VanMMA and VanDiMMA and a worse rheology modifying properties than CinMMA. The results show that VanDiMMA exhibited the most consistent and equidistant diluting character. CinMMA and IBOMA reactive diluents are the least viscous of all studied; therefore, their effect on the viscosity decrease is the most significant. Also, since CinMMA and IBOMA possess high volatility compared to the other compounds (see Figure 12c), their diluting inconsistency is apparent. This is considered as an adverse effect. We used 25 wt % of each reactive diluent to prepare SLA 3D-printed specimens since this mass content is generally used.^55^ Also, the rheological study revealed a possible viscosity decrease for each diluent. The lower the viscosity level, the better and more efficient the 3D printing process since the resin can easily transfer to the printer.

### 3.4 Mechanical and Thermo-Mechanical Properties of Produced Resins

**Figure 9:**
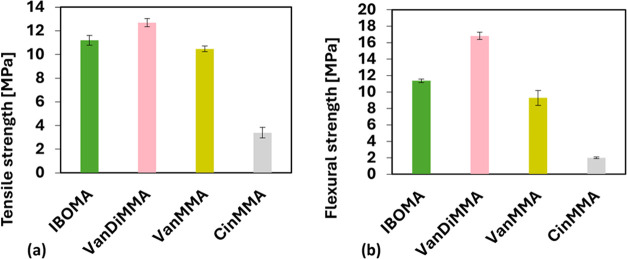
Tensile (a) and flexural (b) strength results of prepared resins.

The mechanical properties of the samples were evaluated through tensile and flexural tests to determine the key parameters relevant to their practical applicability. The tensile stress–strain curves provide insight into the materials’ resistance to deformation and overall ductility, primarily focusing on assessing tensile strength, the maximum stress the material can withstand before failure. Figure 9a presents the measured tensile strength values for each mixture. Among them, VanDiMMA exhibited the highest tensile strength at 12.70 ± 0.34 MPa, which is notable compared to that of the commercially used IBOMA, which reached only 11.19 ± 0.41 MPa. As anticipated, CinMMA displayed the lowest tensile strength, measured at 3.40 ± 0.45 MPa, and VanMMA reached 10.47 ± 0.25 MPa. The exceptional results uncovered for the VanDiMMA diluted resin correspond with previous findings. Since VanDiMMA contains two curable methacrylate groups, higher cross-linking is achieved. Also, VanDiMMA, as an aromatic planar compound, exhibits the dispersion attractive forces that increase in the material, which enhances its tensile properties. On the other hand, CinMMA reached the worst mechanical performance. This outcome is likely caused by the unsaturated double bond in CinMMA’s structure. This double bond prohibits optimal dispersion force formation.

**Figure 10:**
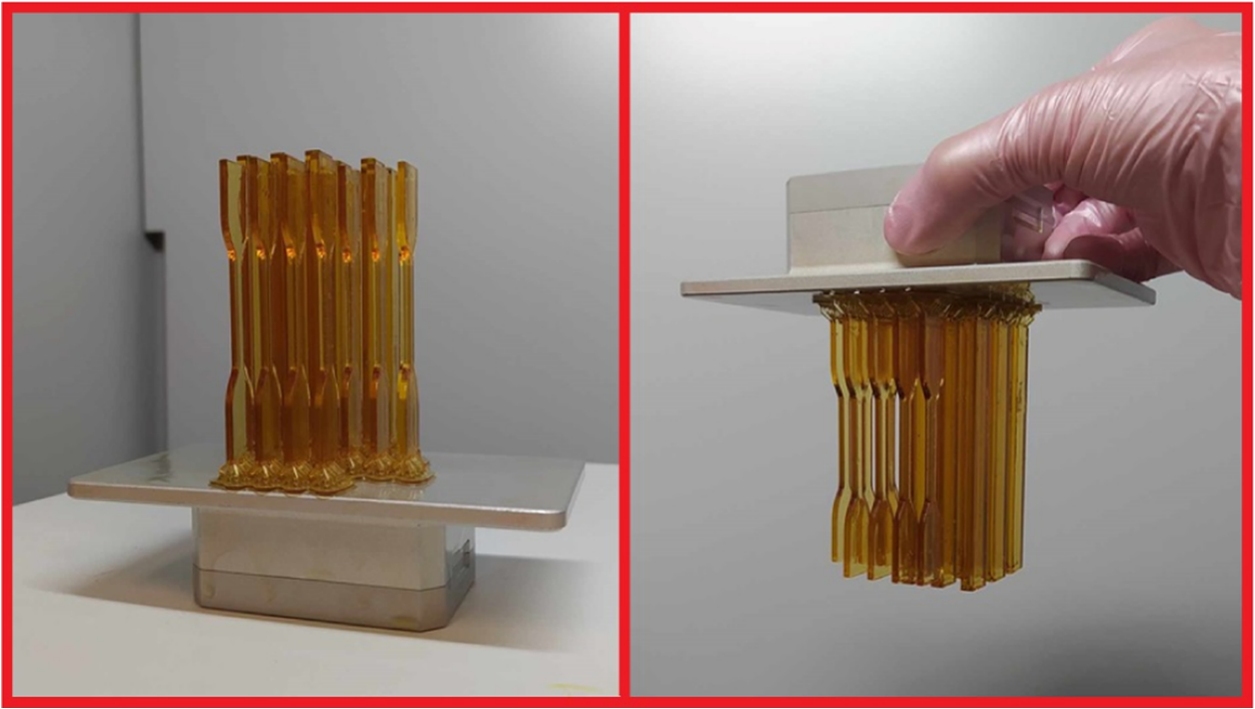
3D-printed specimens for tensile and flexural investigation.

The flexural properties of the samples were evaluated by using a three-point bending test focusing on flexural strength. Figure 9b presents the flexural strength results for each mixture, which are displayed. Once again, VanDiMMA exhibited the highest flexural strength, measured at 16.02 ± 1.84 MPa, significantly outperforming the commercially used IBOMA, which reached 11.35 ± 0.22 MPa. Conversely, CinMMA demonstrated the lowest flexural strength, measuring 2.01 ± 0.10 MPa, and VanMMA reached 9.29 ± 0.91 MPa. These results suggest that the VanDiMMA mixture offers superior bending resistance, aligning with its observed tensile properties compared to other tested resins. The higher flexural and tensile strength of VanDiMMA can likely be attributed to its unique chemical structure, which may allow a greater degree of cross-linking. This increased cross-linking density contributes to enhanced mechanical strength, as demonstrated by its significantly higher flexural strength than the other tested resins. Next to the increased cross-linking density, the aromatic molecular structure plays a significant role in enhancing mechanical properties. The IBOMA’s structure contains a bicyclic aliphatic carbon backbone possessing limited but present molecular movement. On the other hand, the planar aromatic character of VanMMA and VanDiMMA increases the dispersion attractive interactions in the structure. The 3D-printed images of the studied specimens are shown in Figure 10.

**Figure 11:**
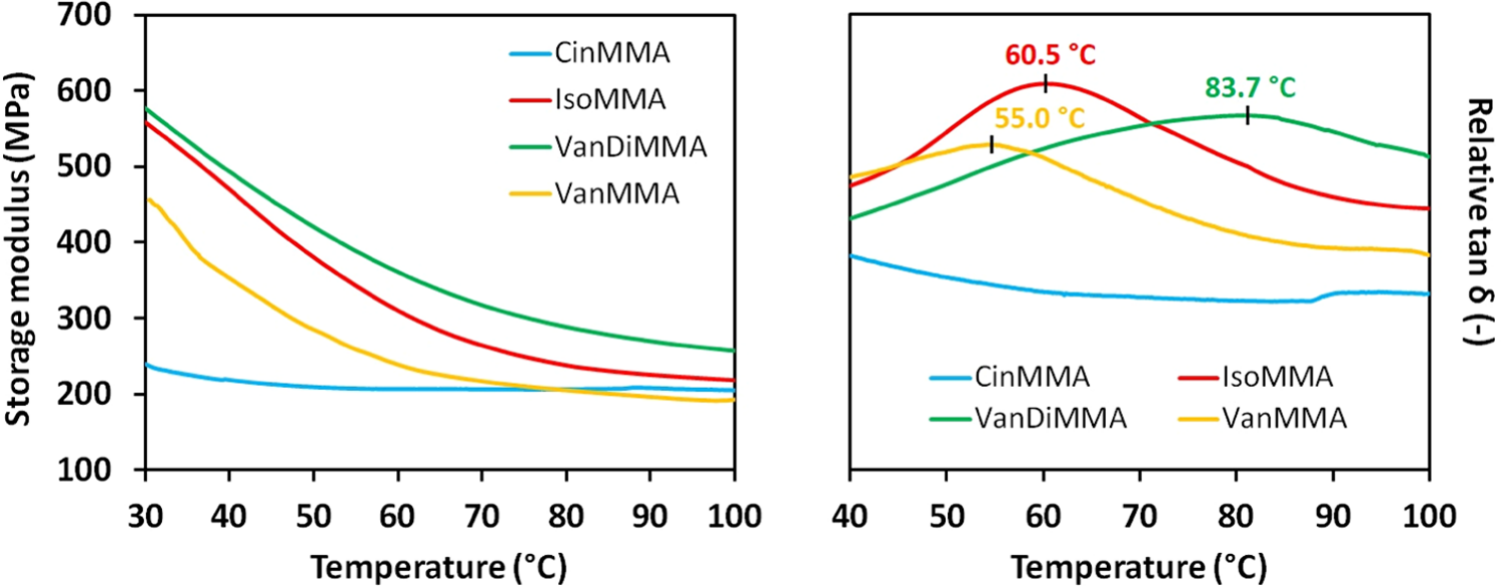
DMA results of cured oil-based resins containing synthesized reactive diluents.

The dynamic mechanical analysis results are displayed in Figure 11. The outcome confirms the trend set during the mechanical analysis. VanDiMMA reached the highest storage modulus at 30 °C (*E*′ = 570 MPa), slightly higher than the mixture with ISOMA (reached *E*′ = 560 MPa at 30 °C). The trend is also reflected in the obtained glass-transition temperatures. VanDiMMA reached the highest *T*_g_ of 83.7 °C compared to other oil-based enhanced resins (IBOMA resin reached *T*_g_ of 60.5 °C and VanMMA exhibited *T*_g_ of 55.0 °C). The lowest dynamic mechanical properties exhibited CinMMA reaching the storage modulus at 30 °C of 240 MPa and the undetected glass-transition temperature at measuring conditions (*T*_g_ < 30 °C). This outcome verifies the mechanical analysis results, providing the same trends for all selected systems. The enhancing properties of VanMMA and VanDiMMA are more promising compared to the previously used commercial and fossil-based additives published in the literature.^46,53^ All additives, cellulose, HEMA, and TMPTA, reached storage modules of 10–500 MPa, with glass-transition temperatures below 25 °C.

**Figure 12:**
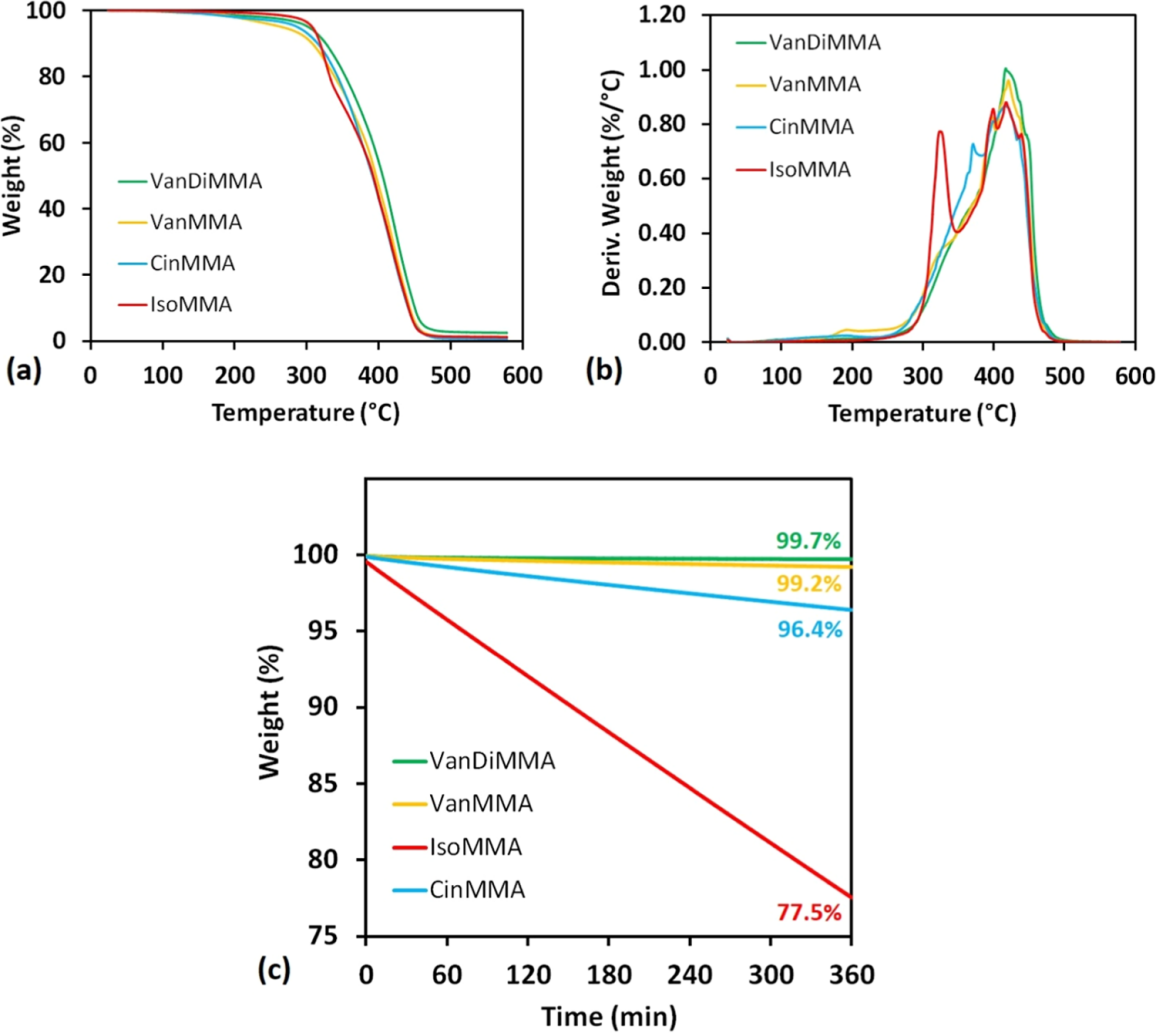
TGA results of volatility and thermal stability of formed oil-based resins: (a) integral TGA curve, (b) derivative TGA curve, and (c) volatility investigation.

The TGA analysis provided information regarding the volatility of the selected reactive diluents used and the thermal stability of oil-based enhanced resins. The results are summarized in Figure 12, where (a) refers to the volatility study and (b, c) illustrates the integral and derivative TGA degradation curve. The volatility study uncovered that ISOMA, as a commercially used reactive diluent, exhibits the highest volatility, causing higher mass loss during the working processes and increasing potential health hazards. The leftover mass content of ISOMA was 77.5% after 360 min at 50 °C. The CinMMA leftover content was 96.4% after the investigation time. VanMMA and VanDiMMA exhibited negligible volatility (99.7% leftover for VanDiMMA and 99.2% leftover for VanMMA). Multiple contributing intermolecular attractive forces, especially the dispersion forces, cause VanDiMMA and VanMMA nonvolatility. Dispersion (London) attractive forces can ideally be expressed in VanDiMMA and VanMMA structures since these molecules are planar.

The thermal degradation results confirmed a strong heat resistivity of the modified vegetable oil. This phenomenon was described in other published articles.^46,52^ We provided the heat-resistant index determination and added it to Table 3 with other TGA results. All of the investigated systems exhibited very similar *T*_S_ values. Although eq 2 uses *T*_max_ for the calculation, the ISOMA-containing system showed a significant degradation signal at lower temperatures. We calculate *T*_S_ using the primary derivative maximum since the system is thermally unstable from this point (the calculation change is marked in Table 3). The heat resistance experiments uncovered that all synthesized systems possess considerably higher thermal stability than the commercially available IBOMA. The poor thermal stability is due to the IBOMA-modified *T*_max_ value (321.1 °C, mentioned in Table 3), indicating significant thermal degradation at a much lower temperature than other systems (*T*_max_ reaching 416–420 °C). The poor thermal stability is a consequence of the IBOMA chemical structure. Due to this outcome, the proposed sustainably produced reactive diluents seem to be an appropriate alternative to the commercial IBOMA. Next to the commercially available IBOMA, our synthesized reactive diluents are also competitive compared to another available diluent, *N*-acryloylmorpholine (ACMO). Though we did not include ACMO in our investigation, its reactive diluting properties were previously published.^56^ ACMO’s apparent viscosity at 25 °C reaches around 10–30 mPa·s. The flexural tests uncovered the same influence when ACMO is used as a reactive diluent. The flexural strength and modulus increased with the content.

**Table 3:** Calculated Results of the TGA Analysis

**thermogravimetric analysis**				
**resin**	***T***_**5**_**(°C)**	***T***_**30**_**(°C)**	***T***_**max**_**(°C)**	***T***_**S**_**(°C)**
**VanMMA**	266.0	363.8	420.6	158.9
**VanDiMMA**	302.5	374.7	416.9	169.5
**CinMMA**	287.4	362.0	416.6	162.7
**IBOMA**	307.8	353.9	321.1*	164.4

* *T*_max_ substituted for partial maximum.

## 4 Conclusions

This paper presents the synthesis, structural verification, and properties study regarding the multicomponent system capable of forming resin suitable for stereolithography 3D printing. The process includes a more sustainable, efficient, and continual approach to minimize the expenses and adverse environmental effects. We synthesized vanillin methacrylate (VanMMA), cinnamyl methacrylate (CinMMA), and vanillyl dimethacrylate (VanDiMMA). The products were structurally verified via NMR, ESI-MS, and FTIR. We used a cheaper, sustainable, and harmless catalyst, potassium acetate, and we also separated the secondary reaction product, methacrylic acid (MA). Furthermore, we used the distilled MA to modify previously epoxidized rapeseed oil to form a curable methacrylated oil precursor. This process achieved more than 90% conversion. The modified oils were also cross-analyzed via NMR, ESI-MS, and FTIR. The synthesized compounds were investigated regarding the rheological modification since they are meant to be reactive diluents. The best-diluting properties exhibited by CinMMA were even better than those of commercial isobornyl methacrylate (IBOMA). Regarding the mechanical and thermo-mechanical properties, VanDiMMA has the best-performing characteristics. This compound succeeded in the highest tensile strength, flexural strength, storage modulus at 30 °C, and glass-transition temperature. VanDiMMA also exhibited a sufficiently low viscosity for reactive diluting purposes. The thermogravimetric analysis also confirmed that VanDiMMA exhibits the least volatility. Based on the recorded results, vanillyl dimethacrylate is an optimal enhancing reactive diluent that was synthesized sustainably and more efficiently compared with recent findings.

## Supporting Information Available

Additional graphical data regarding the structural verification of the synthesized reactive diluents (Figures S1–S20), mechanical properties’ investigation (Figures S21–S22), and thermo-mechanical properties investigation (Figure S23) (PDF)
