# Supplementary material for: Continuous Sustainable Production of Biobased Multicomponent Enhanced Resin for SLA 3D Printing
Source: ACS Mater Au. 2025 Mar 20;5(3):580–92. doi: 10.1021/acsmaterialsau.5c00014 (PMC12082353; doi:10.1021/acsmaterialsau.5c00014)
Supplement: Supplementary file 1 — mg5c00014_si_001.pdf [file mg5c00014_si_001.pdf]

Continuous sustainable production of bio-based multicomponent enhanced resin for SLA 3D printing

Vojtěch Jašek[a*], Otakar Bartoš[a], Veronika Lavrinčíková[a], Jan Fučík[b], Silvestr Figalla[a], Eliška Kameníková[b] and Radek Přikryl[a]

a Institute of Materials Chemistry, Faculty of Chemistry, Brno University of Technology, 61200 Brno, Czech Republic.

b Institute of Environmental Chemistry, Faculty of Chemistry, Brno University of Technology, 612 00 Brno, Czech Republic.

*corresponding author: xcjasekv@vutbr.cz

Table of content

1. **Structural verification of the synthesized reactive diluents**
    - **Figure S1-S4.** Cross-analysis of vanillin methacrylate (Van MMA)
    - **Figure S5-S8.** Cross-analysis of cinnamyl methacrylate (CinMMA)
    - **Figure S9-S12.** Cross-analysis of vanillyl dimethacrylate (VanDiMMA)
    - **Figure S13-S14.** Cross-analysis of epoxidized rapeseed oil (ERO)
    - **Figure S15-S16.** Cross-analysis of methacrylated rapeseed oil (MRO)
    - **Figure S17-S20.** Cross-analysis of vanillyl dimethacrylate of distilled methacrylic acid (MA)
2. **Mechanical investigation data**
    - **Figure S21-S22.** Tensile and flexural stress-strain curves.
3. **Thermo-mechanical investigation data**
    - **Figure S23.** The loss modules of prepared and measured oil-based reactive diluent-containing resins.

1. Structural verification of the synthesized reactive diluents

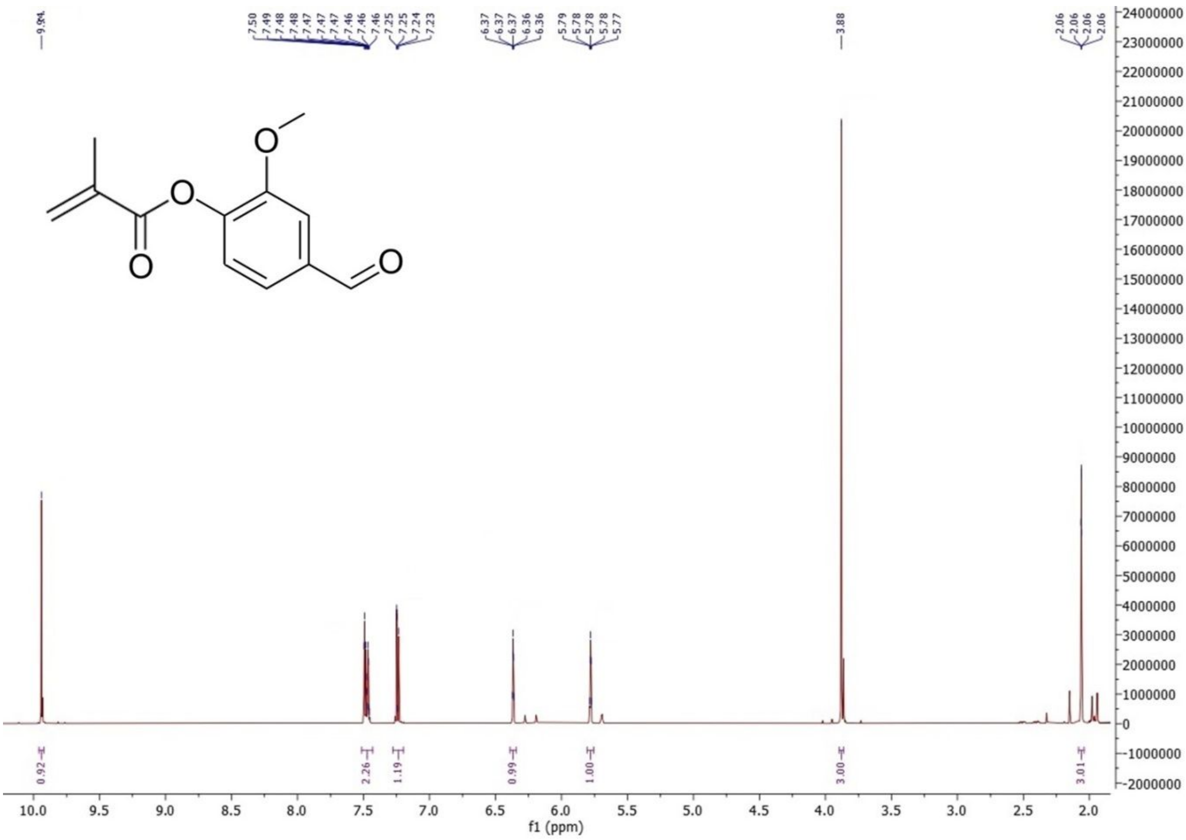

Figure S1. ^{1}H NMR spectrum of vanillin methacrylate (VanMMA).

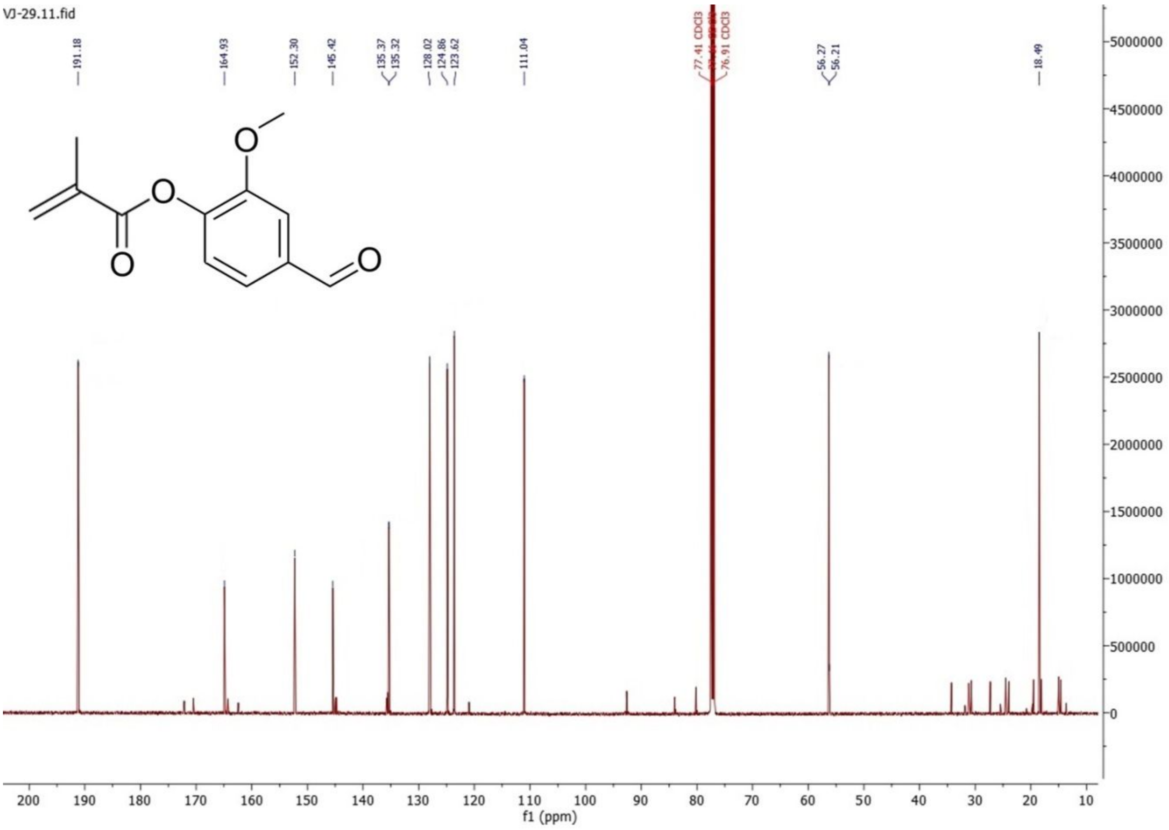

Figure S2. ^{13}C NMR spectrum of vanillin methacrylate (VanMMA).

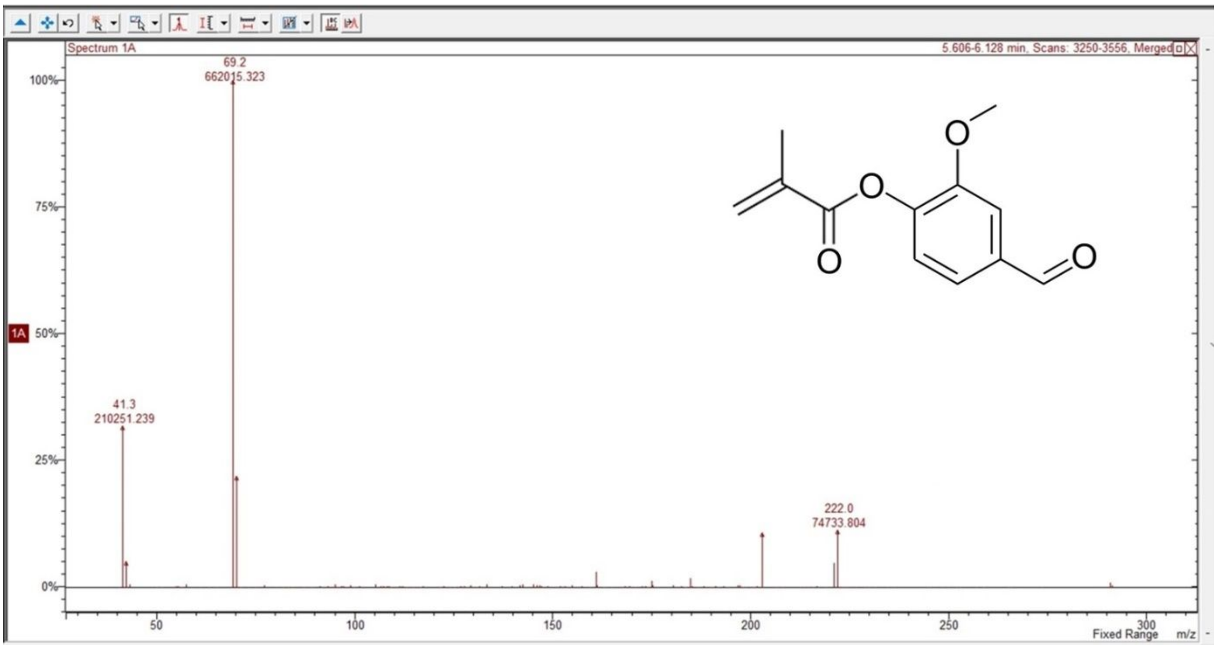

Figure S3. ESI-MS spectrum of vanillin methacrylate (VanMMA).

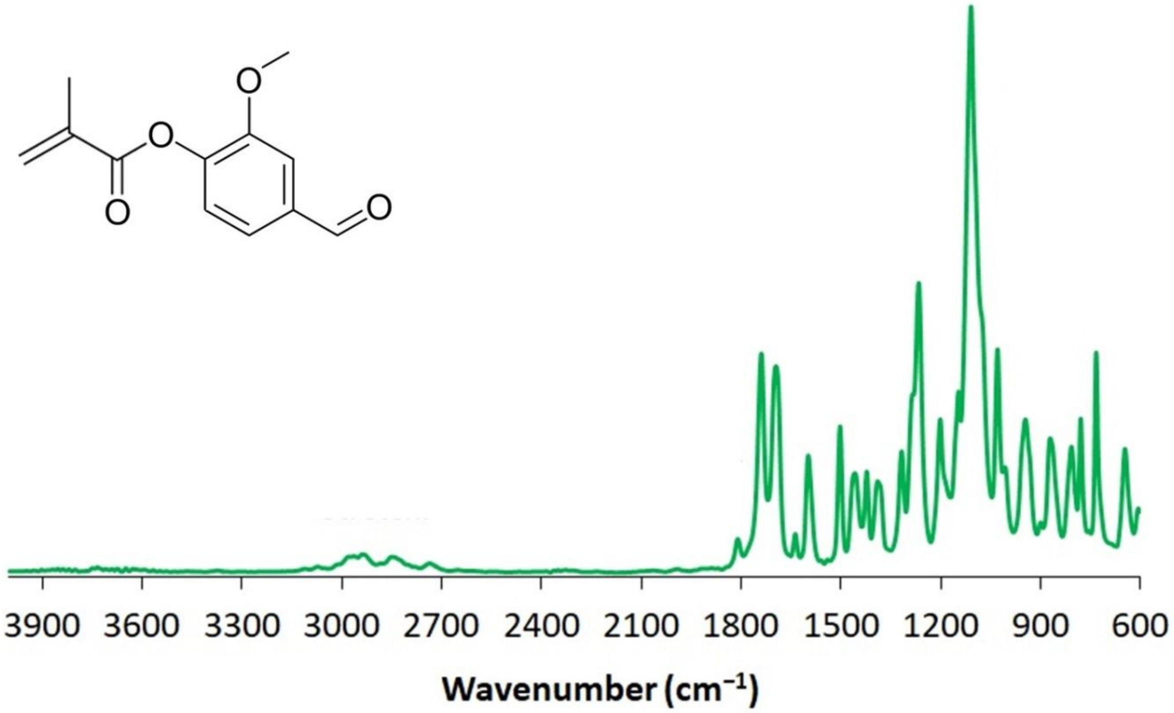

Figure S4. FTIR spectrum of vanillin methacrylate (VanMMA).

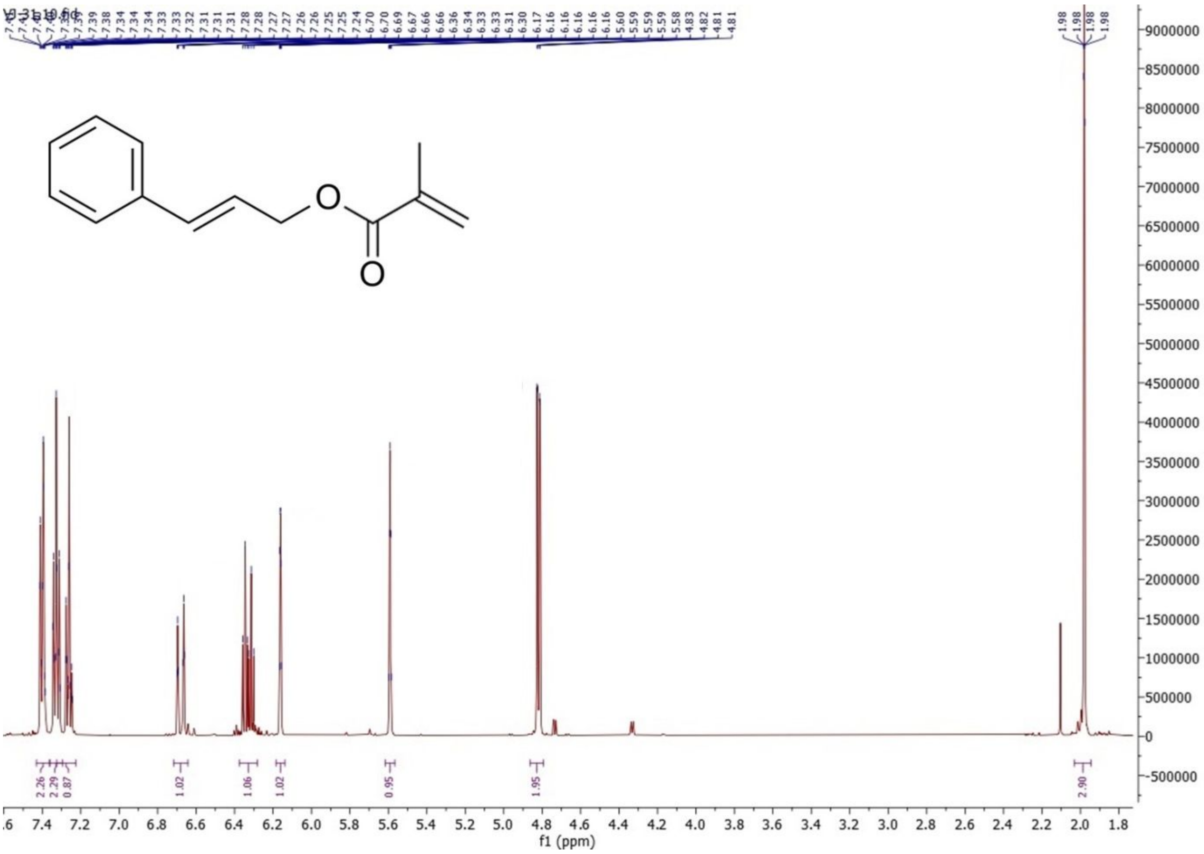

Figure S5. ^{1}H NMR spectrum of cinnamyl methacrylate (CinMMA).

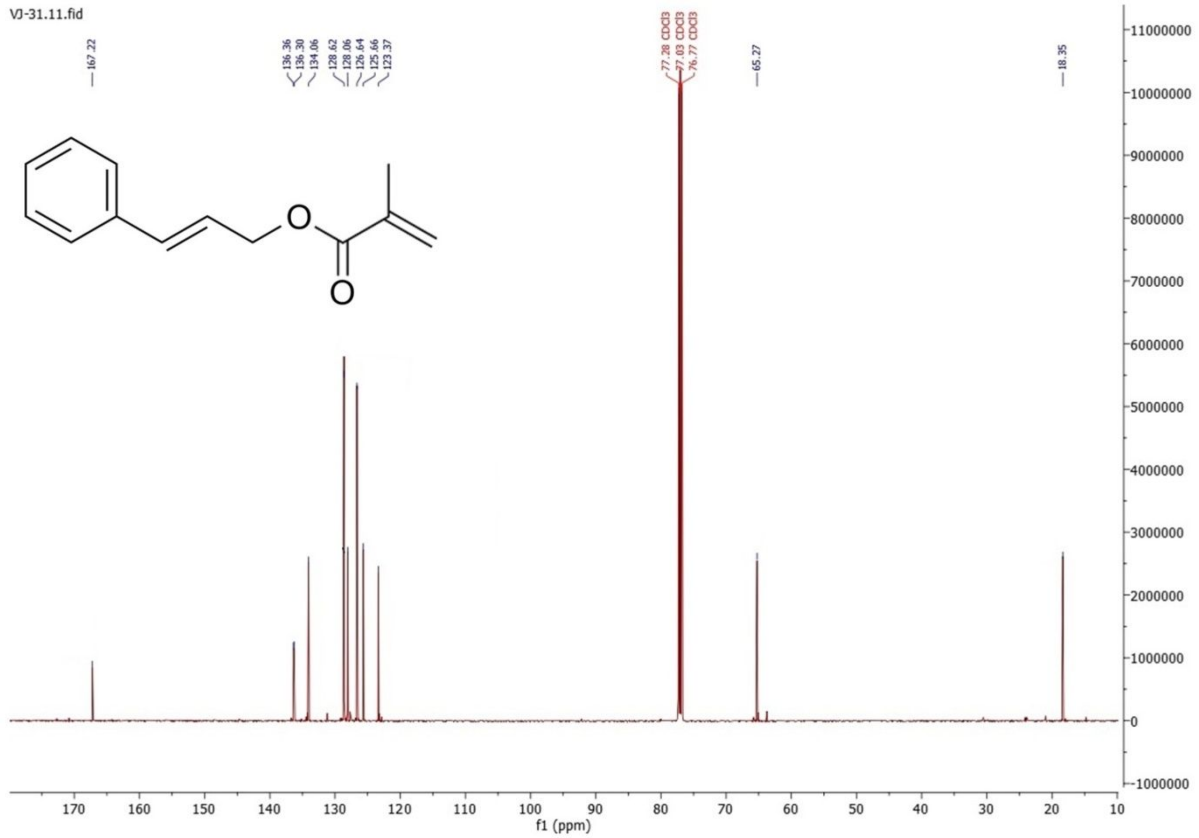

Figure S6. ^{13}C NMR spectrum of cinnamyl methacrylate (CinMMA).

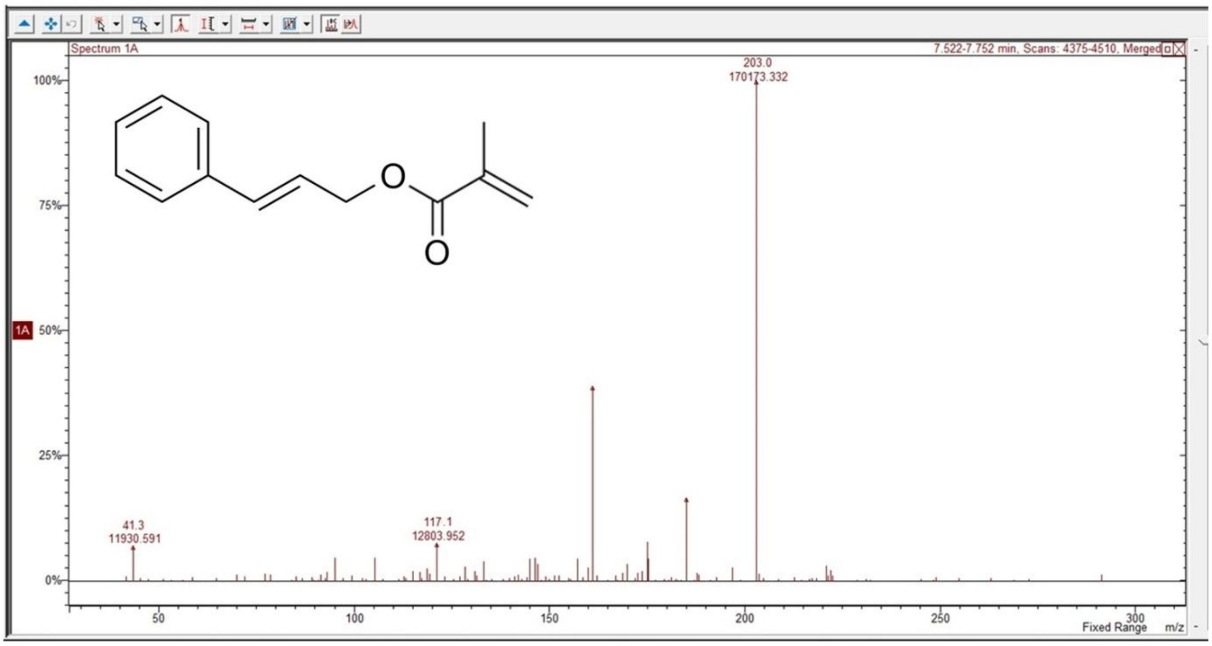

Figure S7. ESI-MS spectrum of cinnamyl methacrylate (CinMMA).

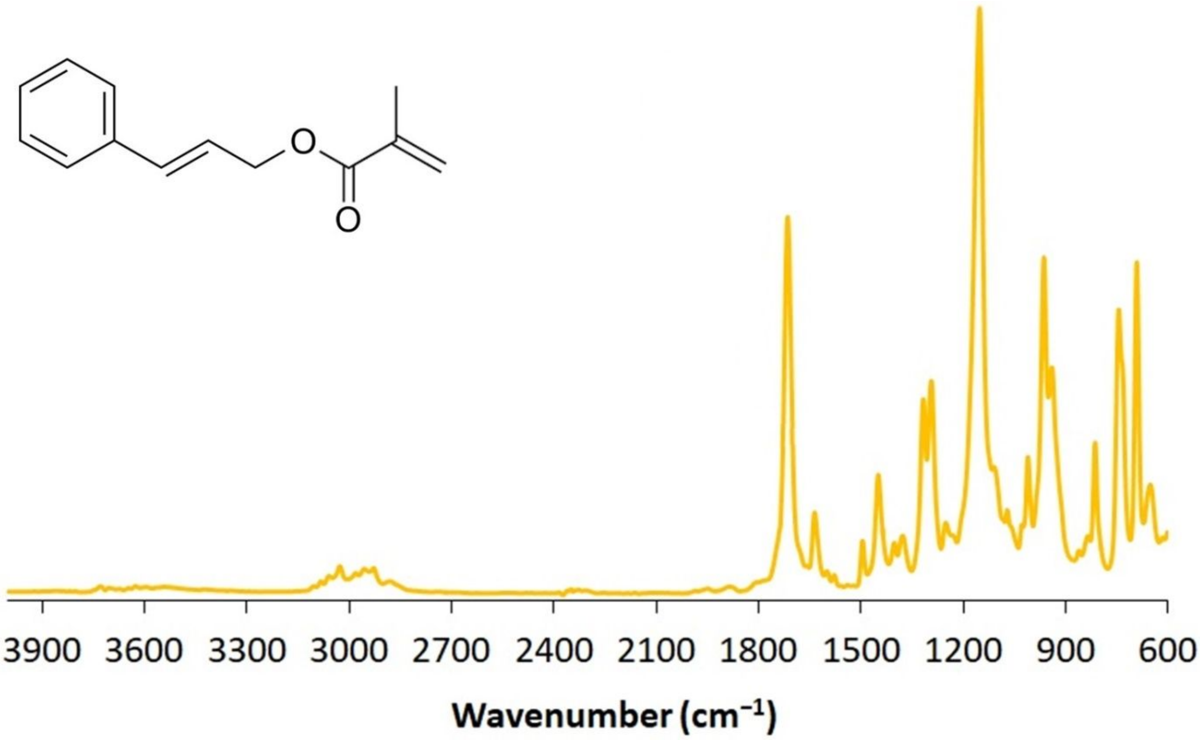

Figure S8. FTIR spectrum of cinnamyl methacrylate (CinMMA).

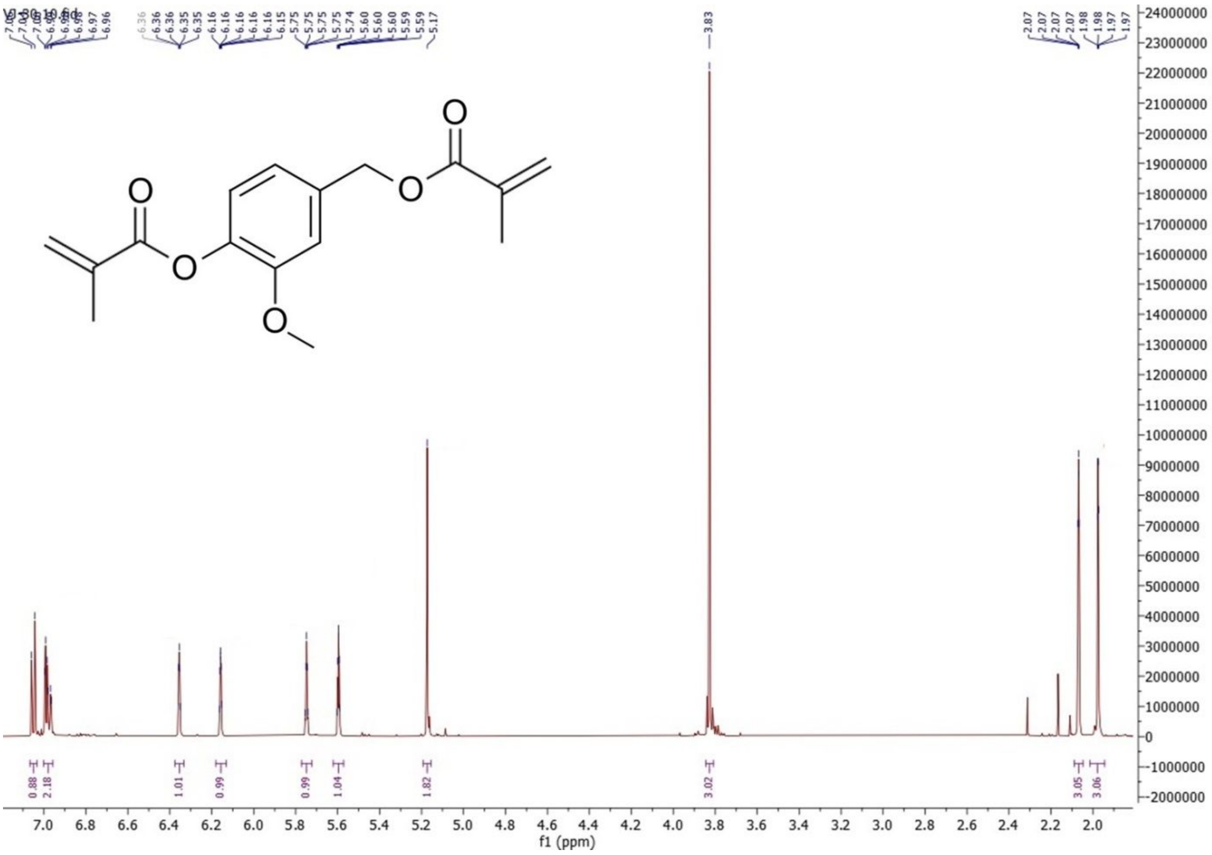

Figure S9. ^{1}H NMR spectrum of vanillyl dimethacrylate (VanDiMMA).

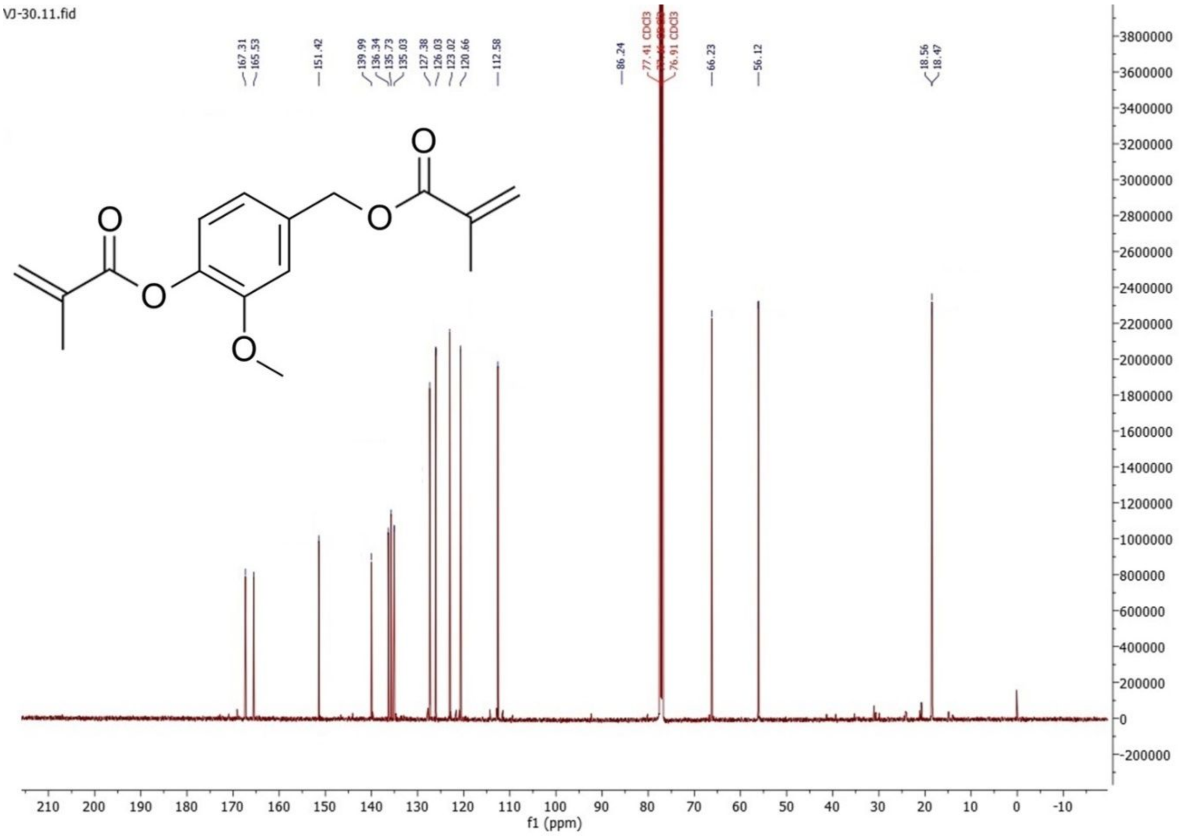

Figure S10. ^{13}C NMR spectrum of vanillyl dimethacrylate (VanDiMMA).

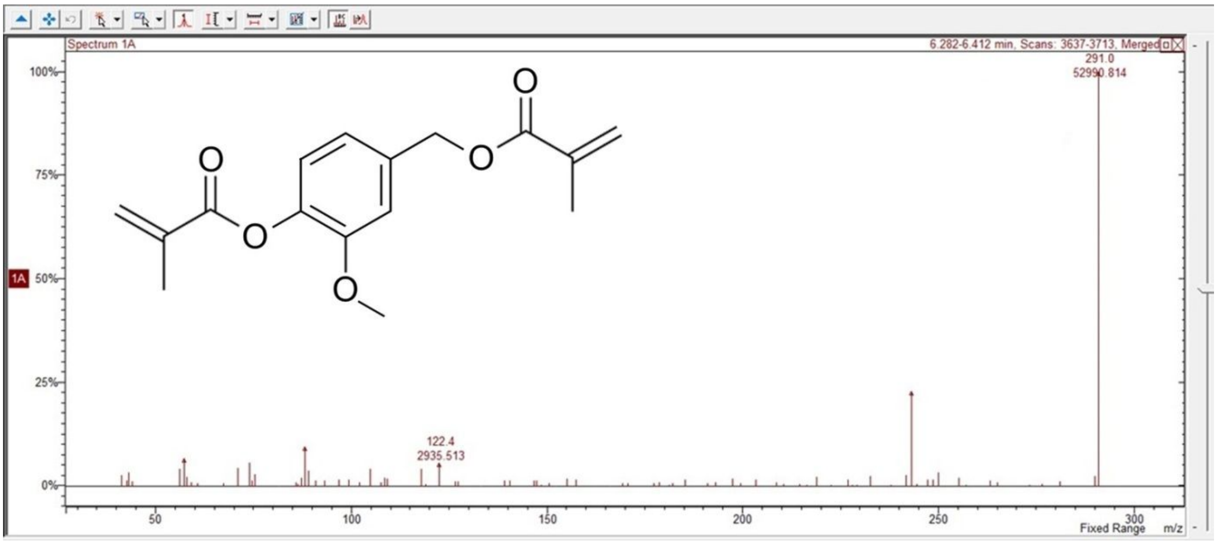

Figure S11. ESI-MS spectrum of vanillyl dimethacrylate (VanDiMMA).

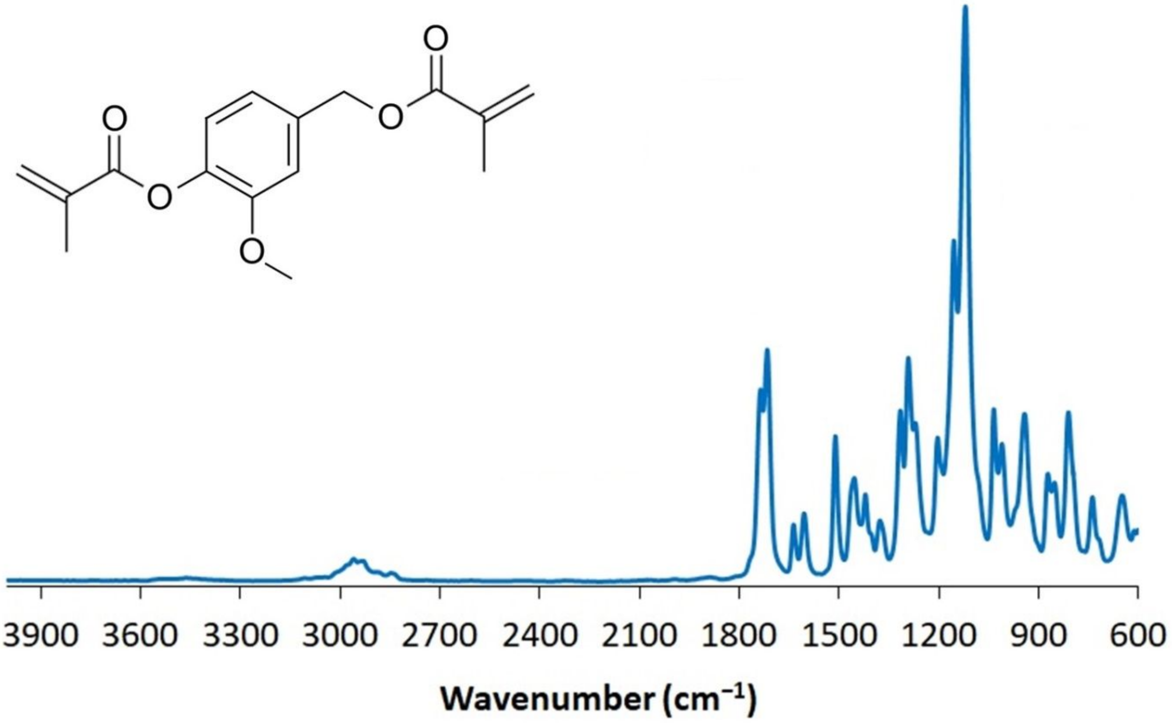

Figure S12. FTIR spectrum of vanillyl dimethacrylate (VanDiMMA).

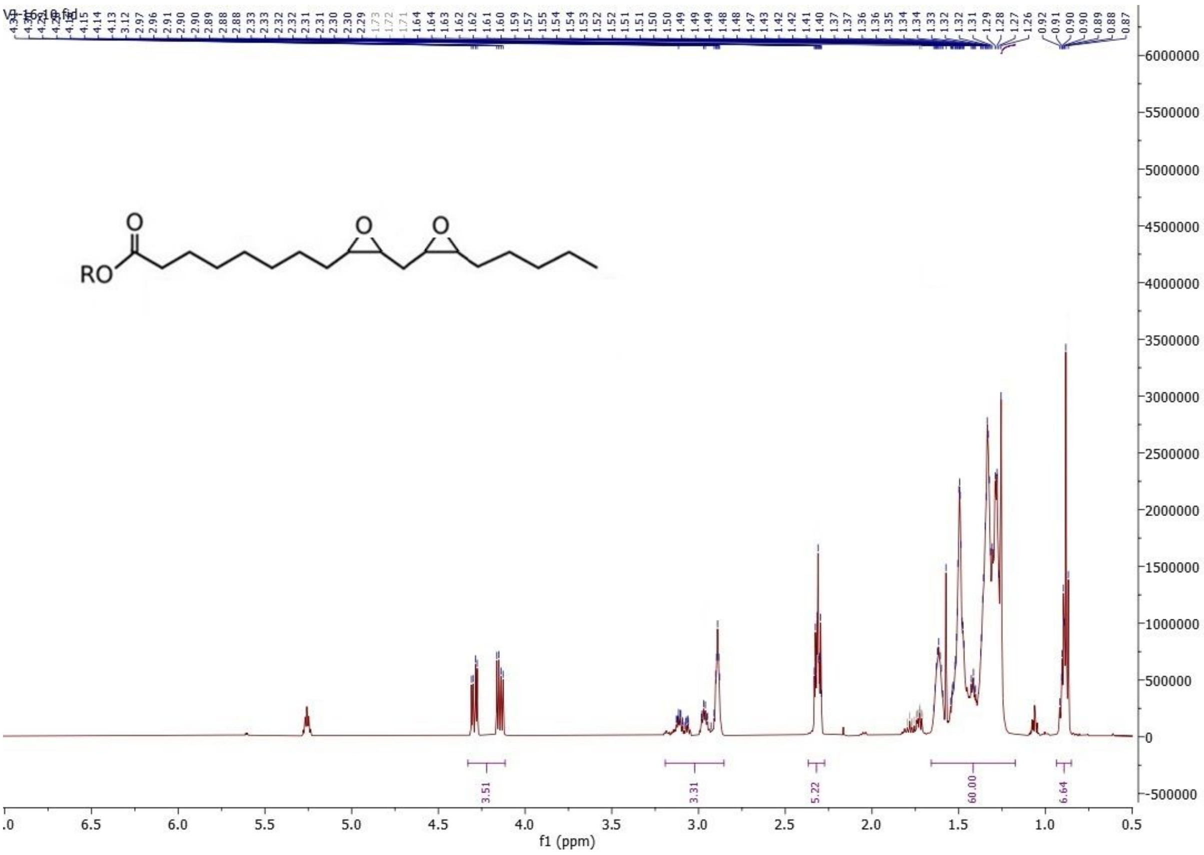

Figure S13. ^1H NMR spectrum of epoxidized rapeseed oil (ERO).

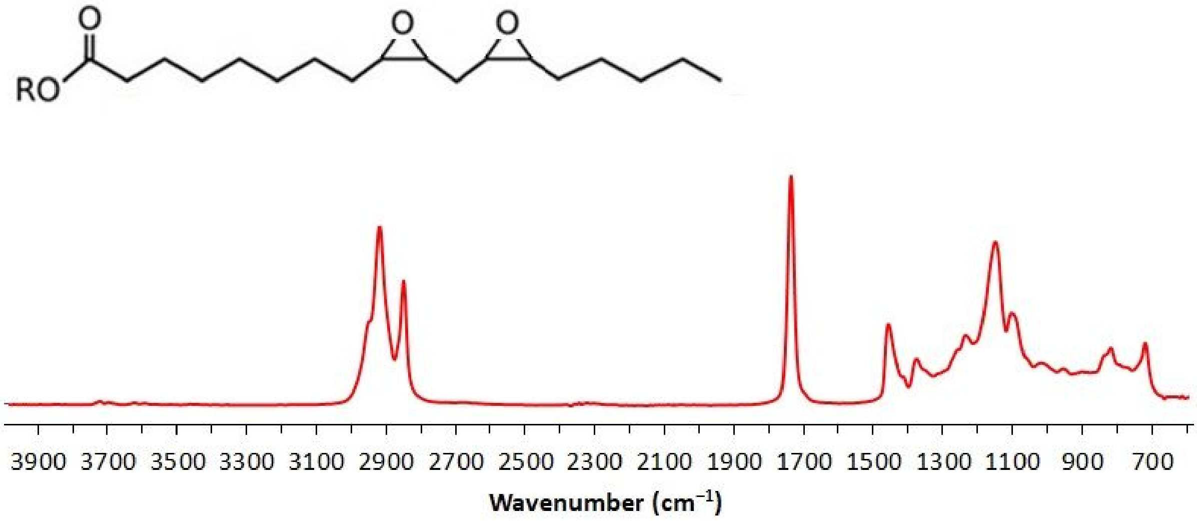

Figure S14. FTIR spectrum of epoxidized rapeseed oil (ERO).

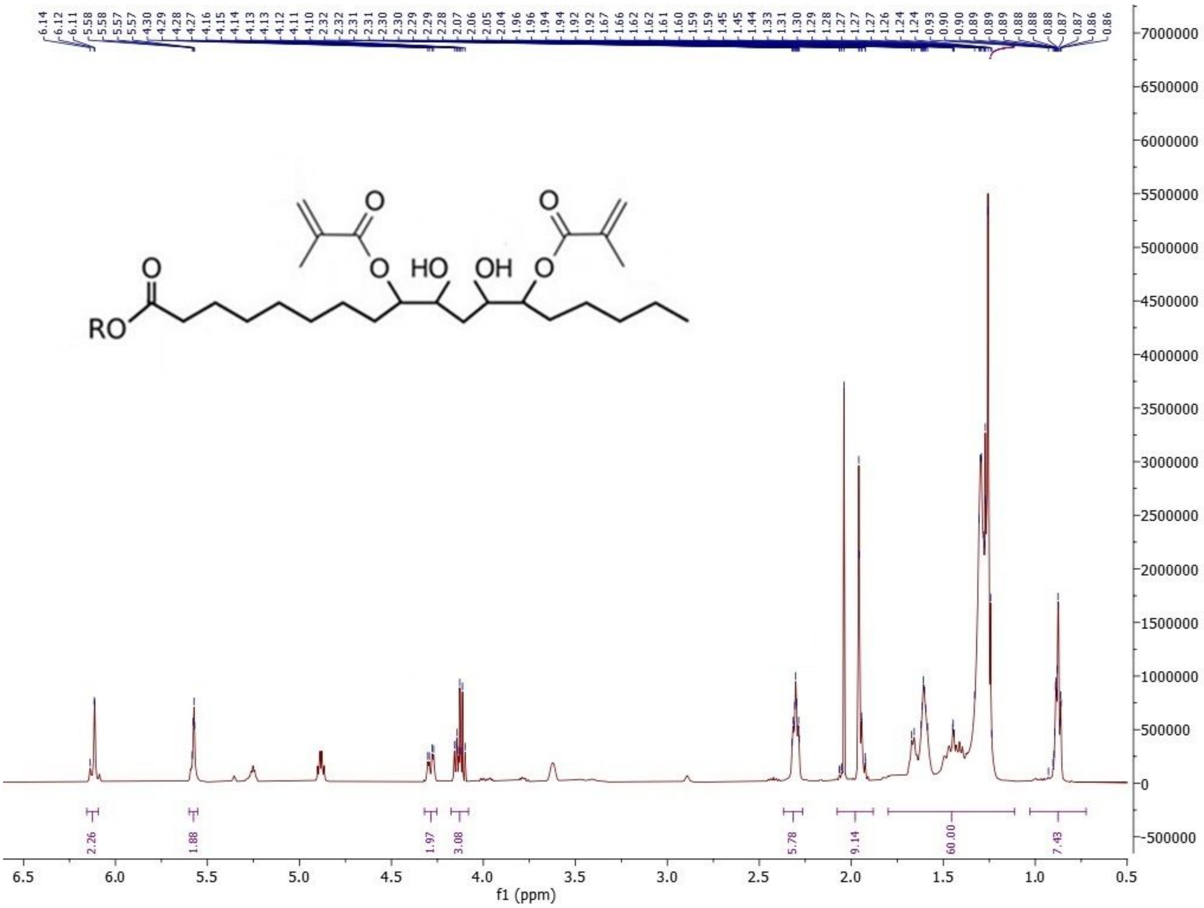

Figure S15. ^{1}H NMR spectrum of methacrylated rapeseed oil (MRO).

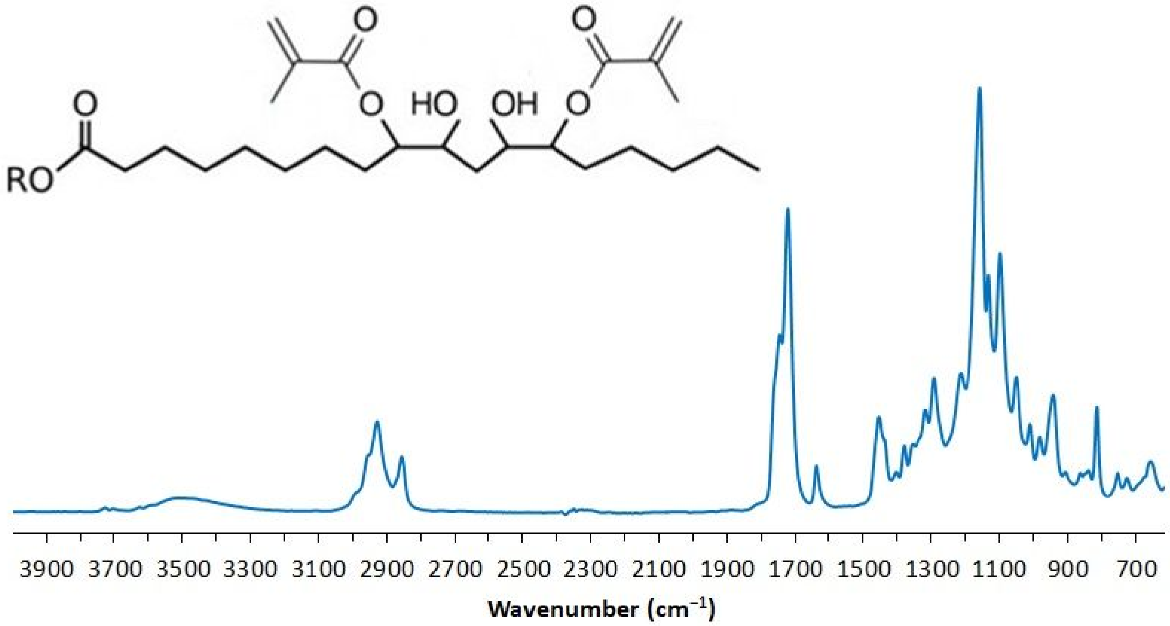

Figure S16. FTIR spectrum of methacrylated rapeseed oil (MRO).

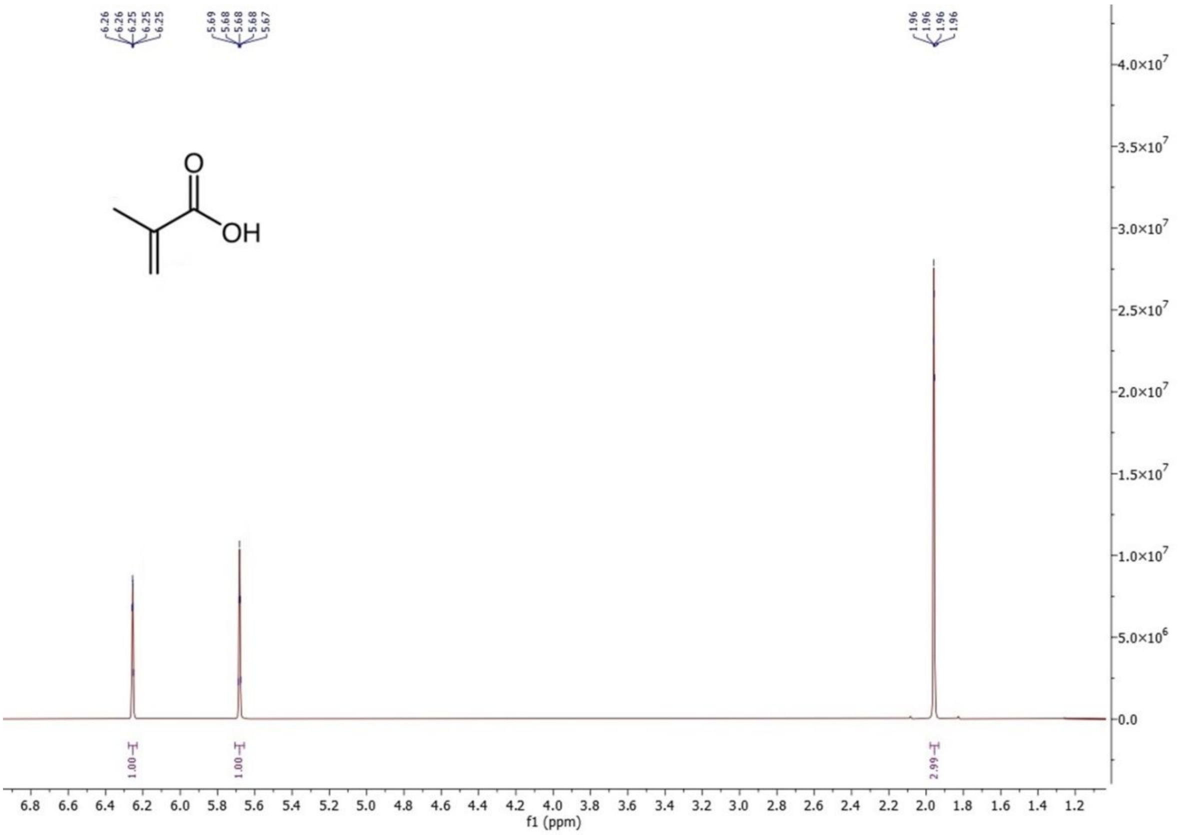

Figure S17. ^{1}H NMR spectrum of methacrylic acid (MA).

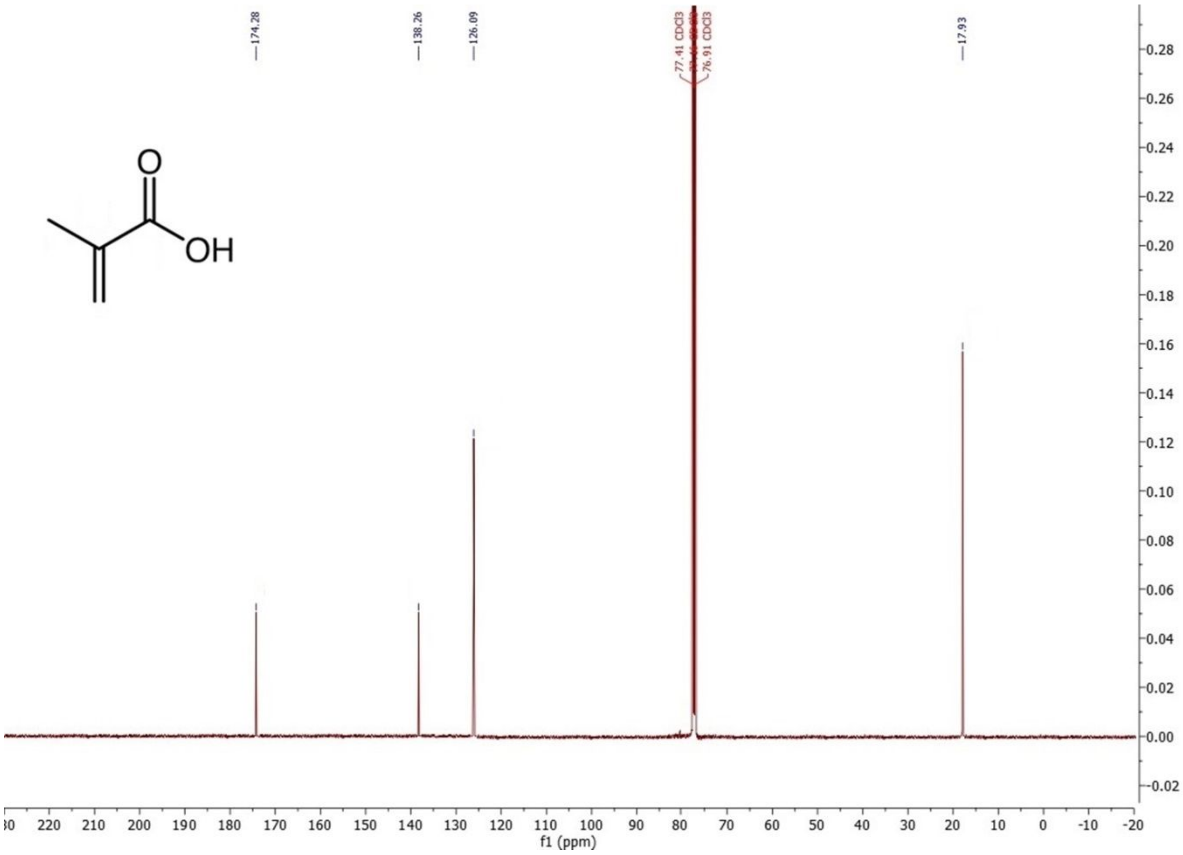

Figure S18. ^{13}C NMR spectrum of methacrylic acid (MA).

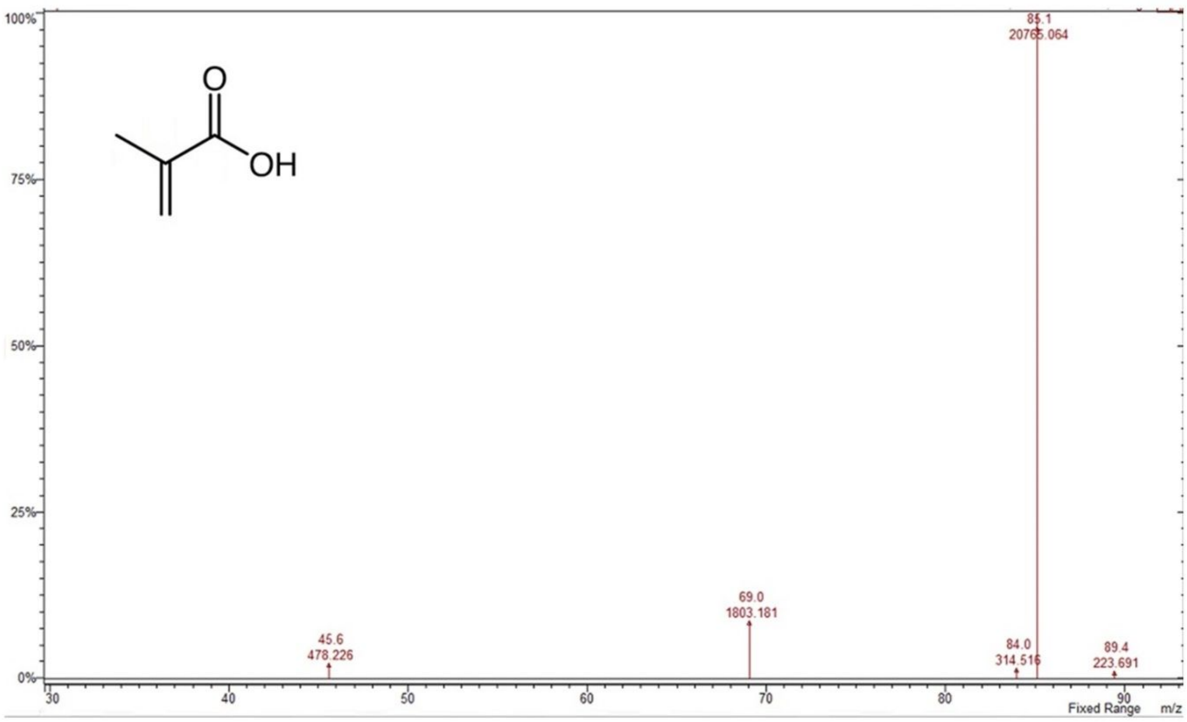

Figure S19. ESI-MS spectrum of methacrylic acid (MA).

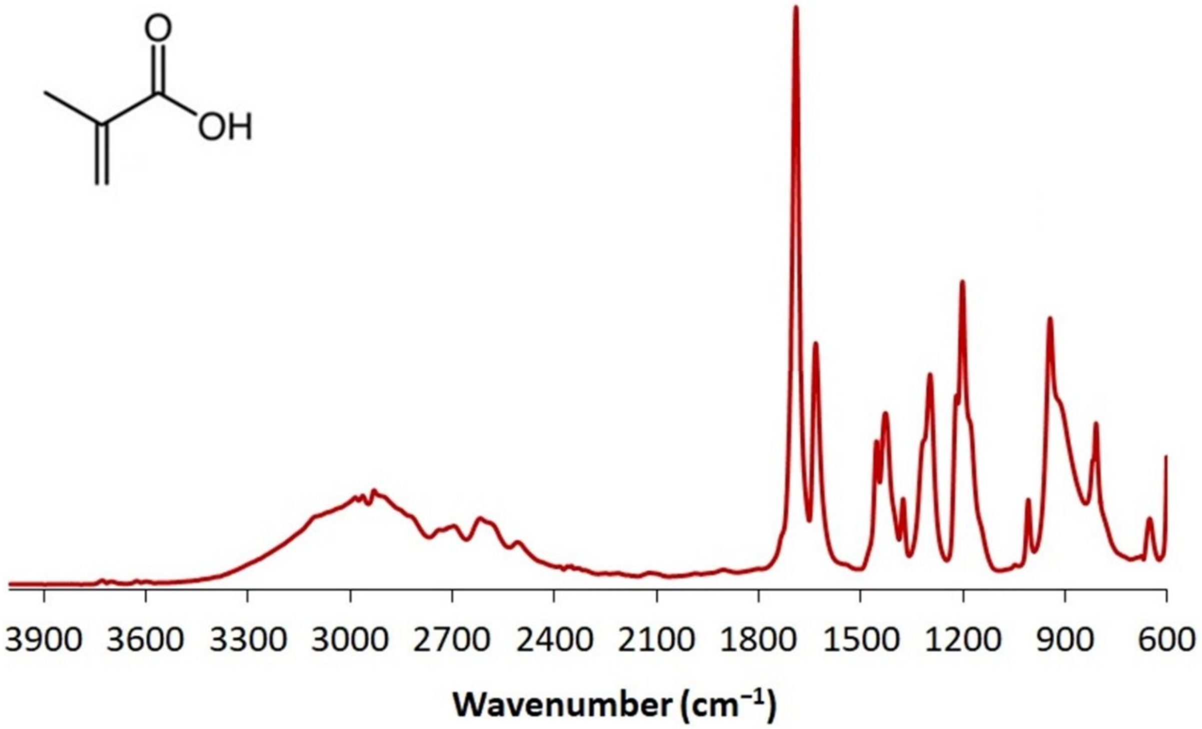

Figure S20. FTIR spectrum of methacrylic acid (MA).

2. Mechanical investigation data

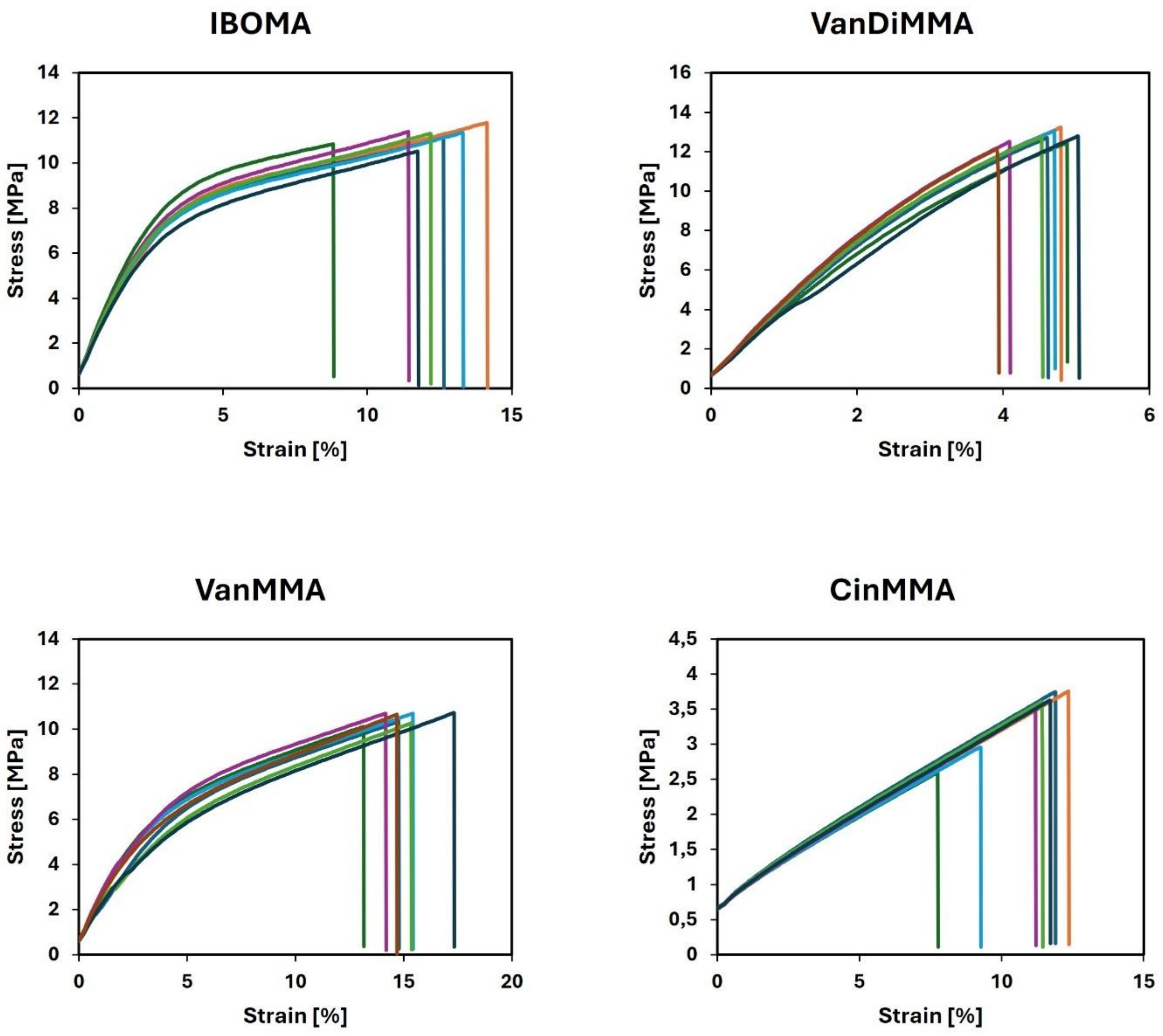

Figure S21. Tensile strength investigation of produced cured oil-based enhanced resins.

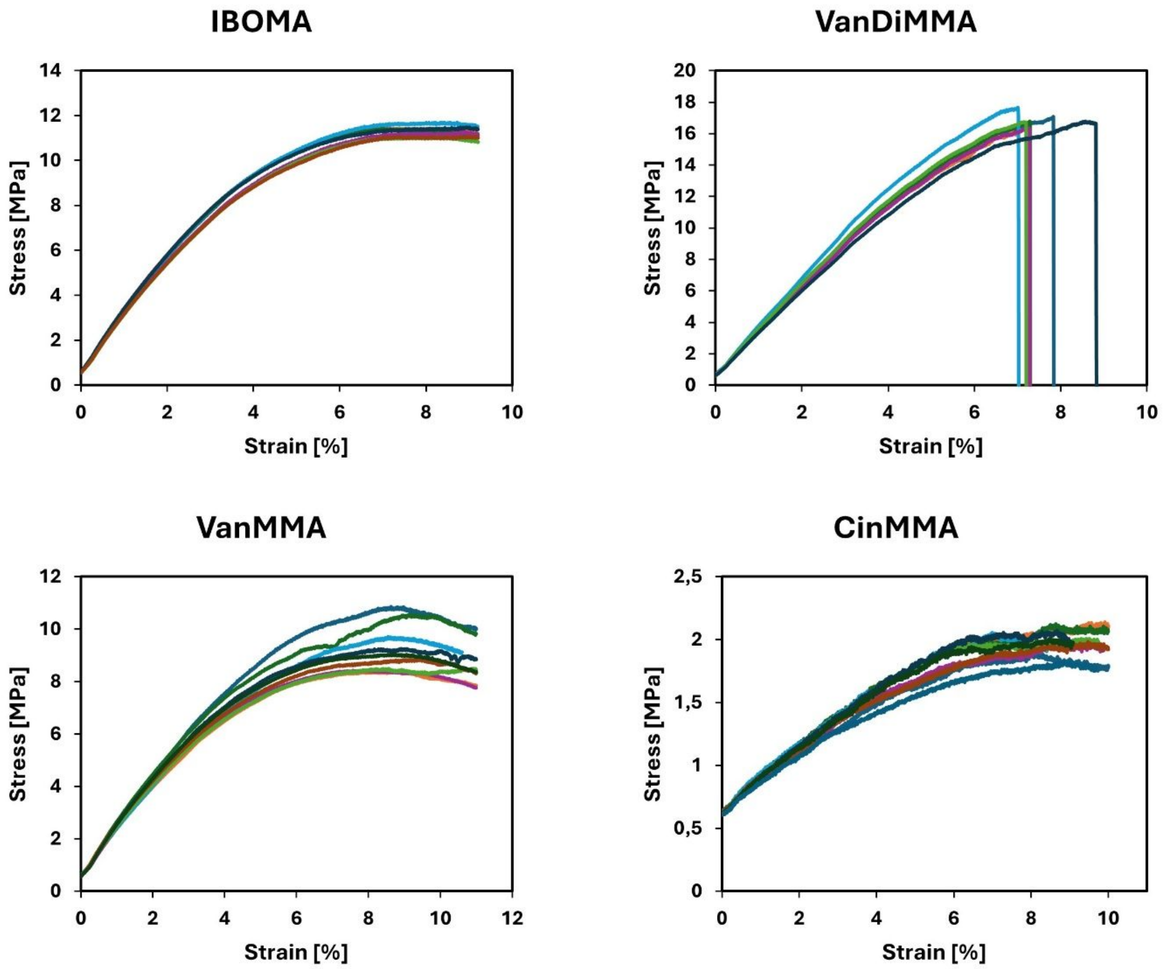

Figure S22. Flexural strength investigation of produced cured oil-based enhanced resins.

3. Thermo-mechanical investigation data

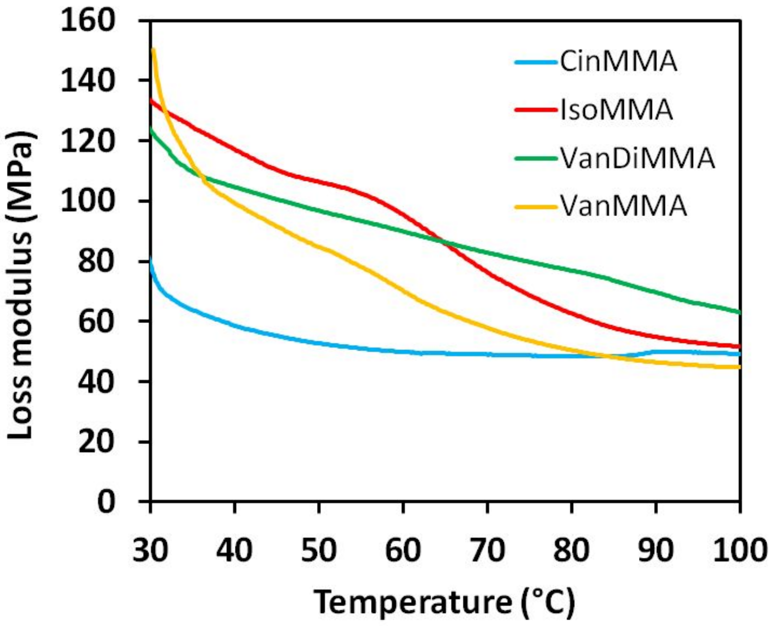

Figure S23. The measured loss modulus of synthesized oil-based resins from DMA analysis.
